# RAB3B Dictates mTORC1/S6 Signaling in Chordoma and Predicts Response to mTORC1‐Targeted Therapy

**DOI:** 10.1002/advs.202415384

**Published:** 2025-03-26

**Authors:** Jianxuan Gao, Jiali Jin, Runzhi Huang, Siqiao Wang, Sihui Song, Yu Zhang, Yongai Li, Jun Lin, Zhengyan Chang, Zongqiang Huang, Wei Sun, Huabin Yin, Dianwen Song, Jianru Xiao, Ping Wang, Tong Meng

**Affiliations:** ^1^ Department of Orthopedics Shanghai Bone Tumor Institute Shanghai General Hospital School of Medicine Shanghai Jiaotong University Shanghai 201620 P. R. China; ^2^ Tongji University Cancer Center Shanghai Tenth People's Hospital School of Medicine Tongji University Shanghai 200072 P. R. China; ^3^ Department of Orthopedics Tongji Hospital School of Medicine Tongji University Shanghai 200065 P. R. China; ^4^ Department of Pathology Shanghai General Hospital School of Medicine Shanghai Jiaotong University Shanghai 201620 P. R. China; ^5^ Department of Pathology Shanghai Tenth People's Hospital School of Medicine Tongji University Shanghai 200072 P. R. China; ^6^ Department of Orthopedics The First Affiliated Hospital of Zhengzhou University Zhengzhou 450052 P. R. China; ^7^ Department of Orthopedics Oncology Changzheng Hospital Navy Medical University Shanghai 200003 P. R. China

**Keywords:** chordoma, DUSP12, mTORC1 signaling, RAB3B, targeted therapy

## Abstract

Chordoma, a rare mesenchymal malignancy, exhibits a high tendency to postoperative recurrence and poor prognosis. To date, its tumorigenic regulatory mechanisms remain elusive, leading to a lack of effective therapeutic targets and drug sensitivity indicators. Here, via transcriptome and proteome analyses, RAB3B is unveiled as a prominent oncogenic regulator in chordoma, with high expression and enhancer‐associated transcriptional activity. Notably, RAB3B ablation attenuated the chordoma cell stemness and malignant biological properties in vivo and in vitro. Through determining the RAB3B‐mediated program in chordoma, it is identified that it enhanced the phosphorylation of S6 specifically at S235/236 and directly bound to S6. Mechanistically, RAB3B physically interacted with phosphorylase DUSP12, and blocked the DUSP12‐mediated dephosphorylation of p‐S6 (S235/236). Pharmacological targeting mTORC1 pathway dramatically impeded the RAB3B‐induced stemness regulation, protein translation, and chordoma tumorigenicity, while RAB3B knockdown desensitized mTORC1 inhibition. In clinic, the combination of RAB3B and p‐S6 suggested a good prognostic value and predicted mTORC1 inhibitors response for chordoma patients. Altogether, this work uncovers RAB3B/DUSP12 as the novel regulators of S6 phosphorylation (S235/236), and suggests the oncogenic and predictive roles of RAB3B/p‐S6 in chordoma, indicating therapeutic potentials of mTORC1‐targeted therapy for advanced chordoma patients with aberrant RAB3B/p‐S6 hyperactivation.

## Graphical abstract

RAB3B is unveiled as a prominent oncogenic regulator in chordoma, which can block the DUSP12‐mediated dephosphorylation of p‐S6 (S235/236). The combination of RAB3B and p‐S6 indicates a good prognostic value and predicts mTORC1 inhibitors response for chordoma patients. This approach enables therapeutic potentials of mTORC1‐targeted therapy for advanced chordoma patients with aberrant RAB3B/p‐S6 hyperactivation.

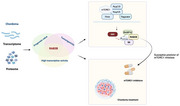

## 1 Introduction

Chordoma is a rare bony and neurologic malignancy with a low‐to‐intermediate grade and locally aggressive nature.^[^ ^1^ ^]^ As chordoma originates from undifferentiated notochord remnants, it mainly affects fused segments of spine, such as occipitocervical and sacrococcygeal regions.^[^ ^2^ ^]^ Therapeutically, the complicated anatomical structures of axial skeletons often make chordoma resection with negative microscopic margins challenging, leading to a high rate of local recurrence.^[^ ^3^ ^]^ Thus, postoperative adjuvant therapies are recommended, whereas the chemo‐ and radio‐resistant nature of chordoma often compromises these therapies.^[^ ^3^, ^4^ ^]^ Additionally, targeted therapies, such as imatinib and sunitinib, only provide limited therapeutic effects.^[^ ^5^ ^]^ Therefore, investigating its tumorigenic mechanism is an urgent need which may provide candidate therapeutic modalities for chordoma.^[^ ^6^ ^]^

Gene somatic mutations are commonly found in chordoma, in which TBXT, CDKN2A, and LYST accounting for a higher proportion.^[^ ^7^ ^]^ TBXT is a diagnostic marker of chordoma and its single‐nucleotide polymorphism (rs2305089), germline duplication and somatic copy‐number gains confer major susceptibility to chordoma.^[^ ^7^, ^8^ ^]^ Thus, targeting TBXT is a candidate option for chordoma treatment. However, as a transcription factor located in cell nucleus, TBXT and its protein brachyury are not easily blocked.^[^ ^9^ ^]^ In addition to TBXT, PI3K signaling components, such as PIK3CA, PTEN, and PIK3R1, can also be mutated in chordoma.^[^ ^7^ ^]^ Moreover, its upstream regulators (EGFR and PDGFRA), along with downstream tuberous sclerosis complex (TSC)/mammalian target of rapamycin complex 1 (mTORC1) signaling, bestow tumorigenic potential to chordoma.^[^ ^10^ ^]^ Given this, PDGFR, EGFR, and mTORC1 inhibitors (imatinib, erlotinib, everolimus, etc) have been applied for chordoma patients in clinic and provide individual responses.^[^ ^5^ ^]^ However, no predictable indicator has ever been identified that limits their individual application.

Nowadays, most sequencing studies of chordoma, including our previous one, mainly focus on the genome.^[^ ^7^, ^11^ ^]^ Nevertheless, in addition to gene mutations, alterations in mRNA and protein also contribute to oncogenesis and cancer progression, which remain elusive in chordoma.^[^ ^12^ ^]^ In this study, via transcriptome and proteome analyses and in‐depth functional studies, we identify RAB3B as a prominent tumorigenic regulator in chordoma with high transcriptional activity and prognostic value. Further mechanistic studies unveil RAB3B as a novel mediator of mTORC1 pathway which enhances the site‐specific modification of S6 (S235/236 phosphorylation) via directly binding to DUSP12 and weakening its dephosphorylated effects on the phosphorylation of S6. Moreover, our results indicate RAB3B/p‐S6 as a candidate response predictor of chordoma patients to mTORC1‐targeted therapies.

## 2 Results

### 2.1 Identification of the Potential Tumorigenic Regulators in Chordoma

As chordoma and nucleus pulposus (NP) both originate from notochord, embryonic NP serves as the appropriate control of chordoma, rather than para‐tumor bone tissue. To identify the potential tumorigenic regulators in chordoma, we performed the transcriptome and proteome analyses comparing chordoma and NP. Via transcriptome analysis between chordoma (*n* = 30) and NP (*n* = 3), a total of 1151 differentially expressed genes (DEGs) were identified with the cutoffs of |log2 fold‐change (FC)| > 2.0, in which 1118 genes were upregulated, and 33 genes were downregulated (Data S1, Supporting Information). The heatmap revealed that MIR5690, SNORD15B, RAB3B were highly expressed in chordoma, along with the generally reported chordoma regulators, such as KRT19, LYST, and EGFR (Figure S1A, Supporting Information). To identify the potential tumorigenic biological functions and signaling pathways in chordoma, Gene Ontology (GO) and Kyoto Encyclopedia of Genes and Genomes (KEGG) analyses were utilized. Via GO analysis, DEGs enriched in biological process (BP) were relevant to positive regulation of cellular catabolic process (Figure S1B, Supporting Information). The significant GO pathways for cellular component (CC) and molecular function (MF) modules were actomyosin and GTPase regulator activity, respectively (Figure S1C,D, Supporting Information). Insulin resistance and endocytosis were identified as the most significant enriched axis in KEGG analysis (Figure S1E, Supporting Information).

In the proteomics analysis between chordoma (*n* = 7) and NP (*n* = 7), a total of 5810 proteins were identified, with a high correlation coefficient for technical replicates (0.965), indicating the technical reliability (Figure S2A, Supporting Information). UMAP revealed the differential distribution of chordoma and NP samples in the batch (Figure S2B, Supporting Information). As for differentially expressed proteins (DEPs), |log2 (FC)| > 0.5 was used as the cutoff. The results revealed 438 highly expressed proteins and 749 lowly expressed proteins between chordoma and NP (Data S1, Supporting Information). In addition to the well‐known chordoma biomarkers (LGALS3 and TBXT), the heatmap also revealed IGKV2, IL13RA2, RAB3B, and MAPK3 highly expressed in chordoma (Figure S2C,D, Supporting Information). BP, CC, and MF module of GO analysis indicated that these DEPs were strongly associated with mRNA processing, splicing complex, and structural constituent of ribosome, while splicesome and ribosome were enriched in KEGG analysis (Figure S2E–H, Supporting Information).

### 2.2 Enhancer‐Associated High Transcriptional Activity of RAB3B in Chordoma

**Figure 1:**
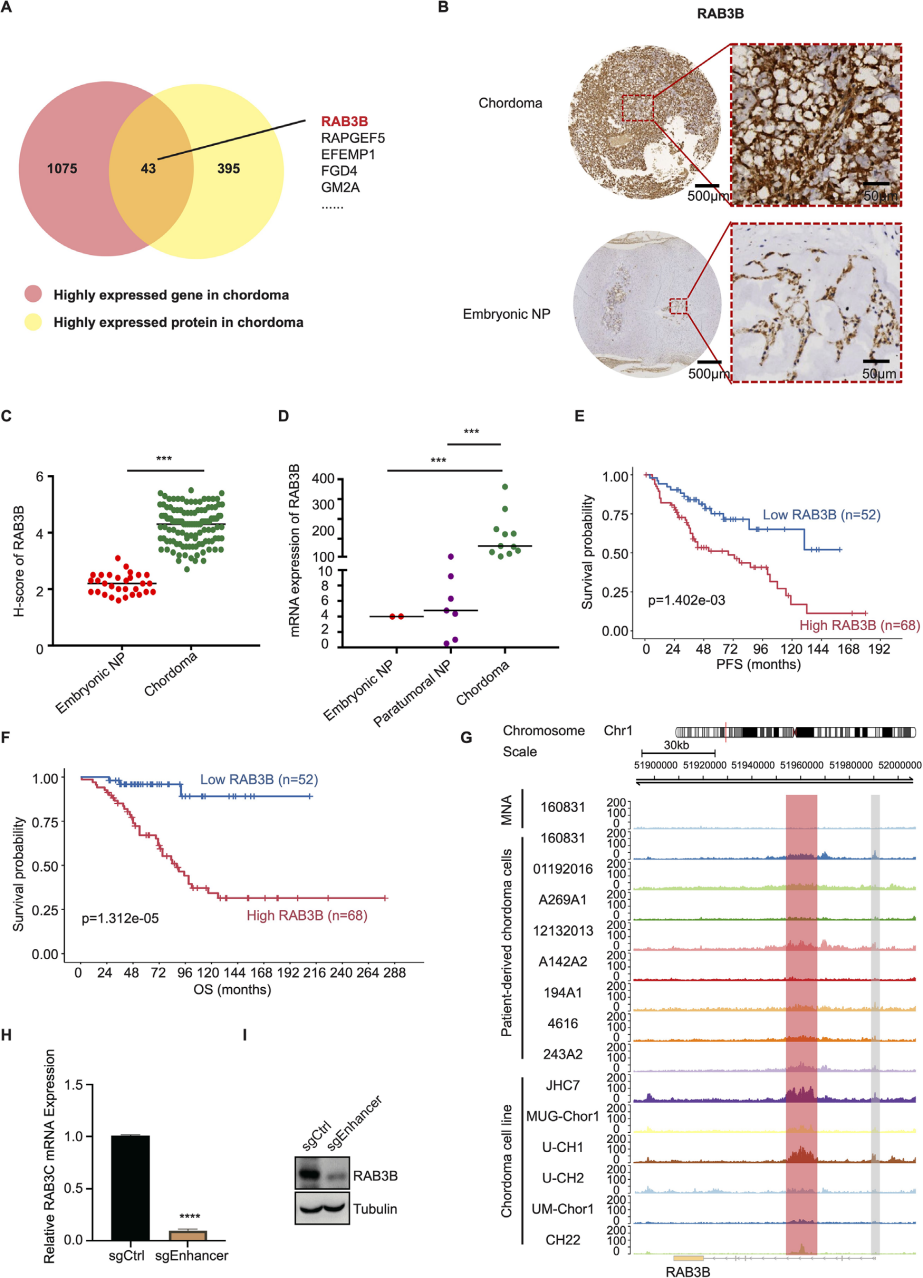
Enhancer‐associated high transcriptional activity of RAB3B in chordoma. (A) The overlapped markers highly expressed in chordoma; (B) Representative images of IHC in chordoma and NP. Scale bar, 50 and 500 µm; (C) Scatter diagram of H‐score of RAB3B in chordoma (*n* = 120) and NP (*n* = 30); (D) Scatter diagram of mRNA expression of RAB3B in chordoma (*n* = 11), embryonic NP (*n* = 2) and paratumoral NP (*n* = 7); Kaplan–Meier analysis of PFS (E) and OS (F) of chordoma patients in low and high RAB3B group; (G) ChIP‐seq of H3K27ac of patient‐derived chordoma cells, cell lines and MNA cells revealed the peaks in the region of RAB3B; mRNA (H) and protein (I) levels of RAB3B in CH22^sgCtrl^ and CH22^sgEnhancer^ cells. ***p < 0.001, ****p < 0.0001.

To explore the potential tumorigenic regulators in chordoma, we identified the overlapped markers between DEGs and DEPs, in which RAB3B ranked first in oncogenic regulators and NF1A served as an important chordoma suppressor (**Figure** **1**A; Figure S3A, Supporting Information). To confirm the RAB3B expression in chordoma, we analyzed the transcriptomic data and tissue microarray (TMA) of chordoma (*n* = 120) and NP (*n* = 30). As for protein levels, we found the overexpression of RAB3B in chordoma, coupled with its low expression in embryonic NP (Figure 1B,C). Compared with embryonic and peritumoral NP, the mRNA levels of RAB3B were significantly higher in chordoma (Figure 1D). In the external validation, the Chordoma Project Supporting Information also revealed highly enriched RAB3B in notochord tissues and chordoma samples (Figure S3B,C, Supporting Information).

The protein levels of RAB3B were classified into high and low‐expression groups based on the median histochemistry score (H‐score). The median H‐score of RAB3B was 4.3, with high RAB3B expression defined as value ≥ 4.3. As described in Figure 1E,F, patients with low RAB3B expression had a longer progression‐free survival (PFS) (*p* = 1.402e–03) and overall survival (OS) (*p* = 1.312e–05).

Next, we explored what induced the high expression of RAB3B in chordoma. The assay for transposase‐accessible chromatin with high throughput sequencing (ATAC‐seq) data of chordoma cell lines (CH22, U‐CH2, and MUG‐Chor1) and NP cells were used to identify the chromatin accessibility and transcription factor occupancy of RAB3B. More peaks were found in the region of RAB3B in chordoma cell lines (Figure S3D, Supporting Information), indicating its high transcriptional activity in chordoma. Additionally, the chromatin immunoprecipitation sequencing (ChIP‐seq) data of histone H3 lysine 27 acetylation (H3K27ac), a chromatin modification associated with active enhancers and promoters, were also compared between patient‐derived chordoma cells/ chordoma cell lines and matched normal adjacency (MNA) cells. As promoters are often located around transcription start sites (TSS), we identified the peaks outside TSS to map enhancers of RAB3B. The results revealed more and consistent peaks in the region of RAB3B, implicating high transcriptional activity of RAB3B in chordoma related to enhancers (Figure 1G). By knocking down of the enhancer and chromatin accessibility regions in RAB3B (chr1: 51955000–51965000), we found the decreased expression of RAB3B in mRNA and protein levels, indicating the enhancer‐associated RAB3B transcriptional activation in chordoma (Figure 1H,I; Figure S3E–G, Supporting Information).

### 2.3 RAB3B Enhances Chordoma Stemness and Tumorigenic Capacity

**Figure 2:**
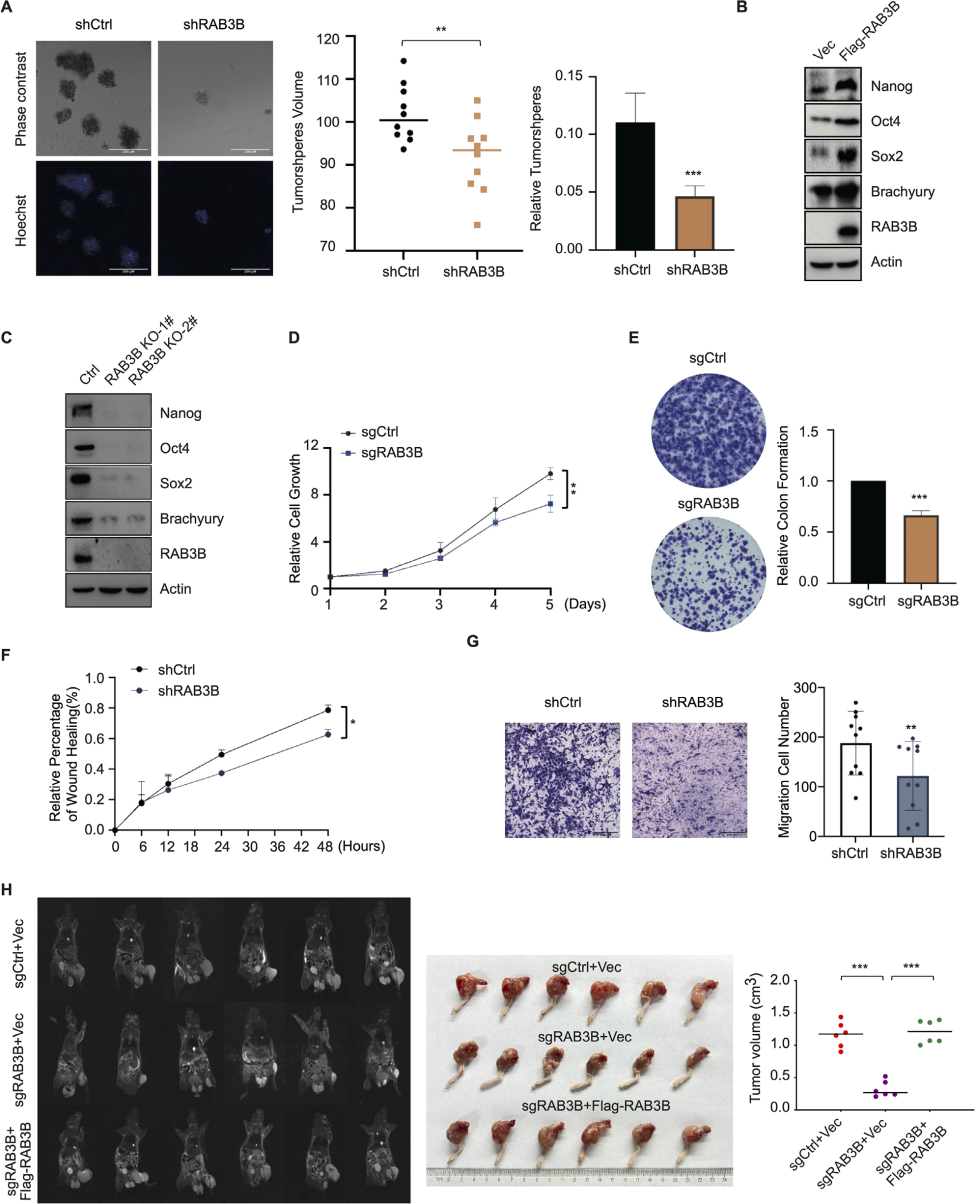
RAB3B enhances chordoma stemness and tumorigenic capacity. (A) Representative images (left), tumorspheres numbers (middle) and volume (right) of cultured tumorspheres in CH22^shCtrl^ and CH22^shRAB3B^ cells (left). Scale bar: 200 µm; (B) Protein levels of stemness markers in the CH22^Vec^ and CH22^Flag‐RAB3B^ cells; (C) Protein levels of stemness markers in the CH22 cells and RAB3B knockout CH22 cells; (D) MTT assay showed a significant time‐dependent inhibition of cell proliferation after knockout of RAB3B in CH22 cells; (E) Representative images of chordoma cell colony formation (left) and number of cell colony formation (right) after knockout of RAB3B in CH22 cells; (F) Wound healing assay showed a significant time‐dependent inhibition of cell migration after knockdown of RAB3B in CH22 cells; (G) Representative images of chordoma cell migration (left) and migration cell number (right) after knockdown of RAB3B in CH22 cells in transwell assay; (H) Representative images of CH22^sgCtrl+Vec^, CH22^sgRAB3B+Vec^, and CH22^sgRAB3B+Flag‐RAB3B^ cells‐bearing nude mice (*n* = 6 for each group). MRI scans (left), tumor‐bearing legs (middle) and tumor volumes (right). *p < 0.05, **p < 0.01, ***p < 0.001.

The chordoma stemness and tumorigenic functions of RAB3B were further evaluated in chordoma. As spheroid formation is an important feature of cancer stemness, we investigated the expression of stemness markers and RAB3B between tumorspheres and adherent non‐sphere cells. The results revealed that high levels of RAB3B, as well as Nanog, Oct4, Sox2, and Brachyury, were verified in the sphere formation assays of chordoma cell lines (Figure S4A–C, Supporting Information). Next, we explored the effects of RAB3B knockdown on sphere formation and found knockdown of RAB3B significantly constrained the tumorsphere formation in chordoma cells (**Figure** **2**A). In the RAB3B‐small‐interfering RNA (siRNA)‐treated chordoma cells, mRNA and protein levels of chordoma stemness markers (TBXT, NANOG, OCT4, and SOX2) were significantly decreased (Figure S4D–F, Supporting Information). Moreover, overexpression and knockout of RAB3B also validated its roles in regulating chordoma stemness markers (Figure 2B,C; Figure S4G,H, Supporting Information). Based on the transcriptomic data of clinical chordoma tissues, RAB3B showed a high correlation with stemness markers (Figure S4I, Supporting Information), such as TBXT (R = 0.85, *p* = 5e–15), OCT4 (R = 0.5, *p* = 0.00016) and NANOG (R = 0.41, *p* = 0.0027).

Regarding cell growth, we applied MTT and clone formation assays in chordoma cells. Knockout of RAB3B significantly impaired the cell proliferation of CH22 in a time‐dependent manner, which was further validated in RAB3B siRNA‐treated chordoma cells (CH22, U‐CH1, U‐CH2, and MUG‐Chor1; Figure 2D,E; Figure S5A–H, Supporting Information). Next, the migratory ability of chordoma cells was evaluated via wound healing and transwell assays. The results showed that RAB3B also mediated the migration of CH22 cells (Figure 2F,G; Figure S5I,J, Supporting Information). We further explored the tumorigenic roles of RAB3B in vivo. Based on the CH22‐bearing tibia model, knockdown of RAB3B markedly decreased the tumor volume (TV) in nude mice and rescue experiment reinforced the chordoma formation (TV of CH22^sgCtrl+Vec^: 1.1634 ± 0.1984 cm^3^; TV of CH22^sgRAB3B+Vec^: 0.3215 ± 0.1238 cm^3^; TV of CH22^sgRAB3B+Flag‐RAB3B^: 1.2096 ± 0.1805 cm^3^; Figure 2H). To evaluate the roles of other RAB3 members in the chordoma stemness and tumorigenic functions, we found that RAB3A, RAB3C, and RAB3D regulated the proliferation of CH22 cells, whereas they did not control the expressions of stemness markers, such as TBXT, NANOG, OCT4 and SOX2 (Figure S6, Supporting Information). Taken together, these results indicate that RAB3B may serve as a pivotal oncogenic driver of chordoma.

### 2.4 RAB3B Regulates mTORC1 Signaling via Controlling Site‐Specific S6 Phosphorylation (S235/236)

**Figure 3:**
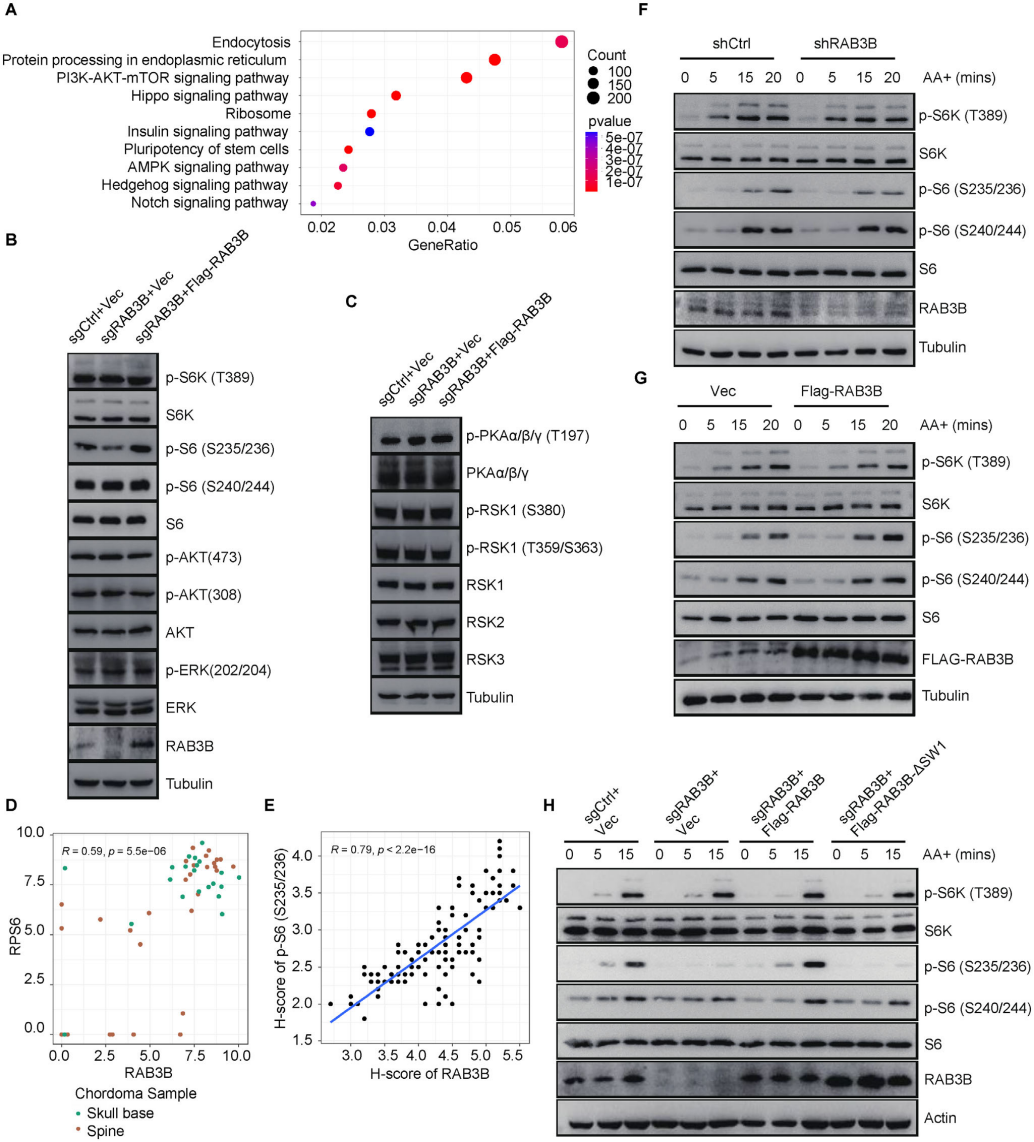
RAB3B regulates mTORC1 signaling via controlling site‐specific S6 phosphorylation (S235/236). (A) KEGG analysis of DEGs between RAB3B wildtype and knockdown CH22 cells; WB analysis to assess the protein levels of PI3K‐AKT‐mTOR signaling (B) and phosphorylase kinases of S6 (C) in CH22^sgCtrl+Vec^, CH22^sgRAB3B+Vec^, and CH22^sgRAB3B+Flag‐RAB3B^ cells; (D) The correlation between RAB3B and RPS6 based on the transcriptome data of chordoma; (E) The correlation of H‐score between RAB3B and p‐S6 (S235/236) based on the TMA of chordoma; (F) WB analysis to assess the protein levels of mTORC1 signaling in CH22^shCtrl^ and CH22^shRAB3B^ cells under AA stimulation; (G) WB analysis to assess the protein levels of mTORC1 signaling in CH22^Vec^ and CH22^Flag‐RAB3B^ cells under AA stimulation; (H) WB analysis to assess the protein levels of mTORC1 signaling in RAB3B knockout, rescue and GTPase (ΔSW1) deletion CH22 cells under AA stimulation.

To unveil the regulatory mechanisms of RAB3B in chordoma cells, RNA‐seq was used to investigate the downstream pathways in RAB3B wildtype and knockdown CH22 cells. The RAB3B‐related genes were enriched in proteasomal protein catabolic process, cell‐substrate junction, and GTPase regulator activity for GO analyses of BP, CC, and MF (Figure S7A–C, Supporting Information). Of interest, in addition to the well‐known function of GTPase activity and endocytosis for RAB GTPases, RAB3B was highly related to PI3K‐Akt‐mTOR signaling (**Figure** **3**A).

PI3K‐AKT‐mTOR signaling plays a prominent role in tumor cell biology and includes a number of components. To elucidate the RAB3B associated PI3K‐AKT‐mTOR regulation, we examined several key markers of PI3K‐AKT‐mTOR signaling (ERK, phospho‐ERK, AKT, phospho‐AKT S473, S6K1, phospho‐S6K1 T389, S6, phospho‐S6 S235/236, phospho‐S6 S240/244) in RAB3B knockout and rescue experiments. The results revealed that p‐S6 (S235/236), but not other markers, was significantly decreased in CH22^sgRAB3B+Vec^ cells (Figure 3B). As such, similar results were identified in RAB3B siRNA‐treated CH22 and U‐CH2 cells (Figure S7D–F, Supporting Information). As ribosomal S6 kinases (RSKs) specifically phosphorylate S6 at S235/236, we further explored additional kinases in modulating RAB3B‐regulated pS6 (235/236) and found that RAB3B did not regulate the expression of RSK1/2/3 and PKAα/β/γ (Figure 3C; Figure S7G, Supporting Information).

Based on our transcriptomic data of chordoma samples, we investigated the correlation between RAB3B and key markers of mTORC1 signaling. The results showed a high correlation of RAB3B with MTOR (R = 0.65, *p* = 2e–07), RPS6K1 (R = 0.83, *p* = 6.5e–14) and RPS6 (R = 0.59, *p* = 5.5e–06) (Figure 3D; Figure S7H, Supporting Information). In the chordoma tissue microarray, a high correlation was also identified between RAB3B and p‐S6 (S235/236, *p* < 2.2e–16) (Figure 3E; Figure S7I, Supporting Information). These results indicated that RAB3B controlled the site‐specific phosphorylation of S6 at S235/236.

As ribosomal protein S6 is the key downstream substrate of mTORC1 activated by intra‐and extra‐cellular signals, we further investigated the roles of RAB3B in the activation of mTORC1 signaling. We generated stable CH22 cell lines with RAB3B knockdown and overexpression. One hour starvation efficiently reduced pS6 signals in CH22 cells and CH22^shCtrl^ cells displayed a stronger ability to phosphorylate S6 (S235/236) than CH22^shRAB3B^ cells upon amino acid (AA) treatment (Figure 3F; Figure S7J, Supporting Information). Consistently, RAB3B overexpression also induced a strongly elevated S6 phosphorylation (S235/236) under AA restimulation (Figure 3G).

As GTPase activity is an important feature of RABs, to explore if the GTPase activity is required to regulate RAB3B associated mTORC1 signaling, we generated RAB3B mutants with deletions in either the switch I domain (RAB3B‐ΔSW1) or the switch II domain (RAB3B‐ΔSW2) to disrupt GTPase activity. Our results showed compared to wild‐type RAB3B, reconstituted RAB3B‐ΔSW1 or RAB3B‐ΔSW2 in RAB3B knock‐out cells attenuated amino acids‐induced phosphorylation of S6, but not S6K (Figure 3H; Figure S7K, Supporting Information). These results suggest that the switch I and switch II domains, essential for RAB3B GTPase activity, play a critical role in regulating mTORC1 signaling via controlling the site‐specific S6 phosphorylation (S235/236).

### 2.5 RAB3B Interacts with DUSP12 and Weakens its Dephosphorylating of p‐S6

**Figure 4:**
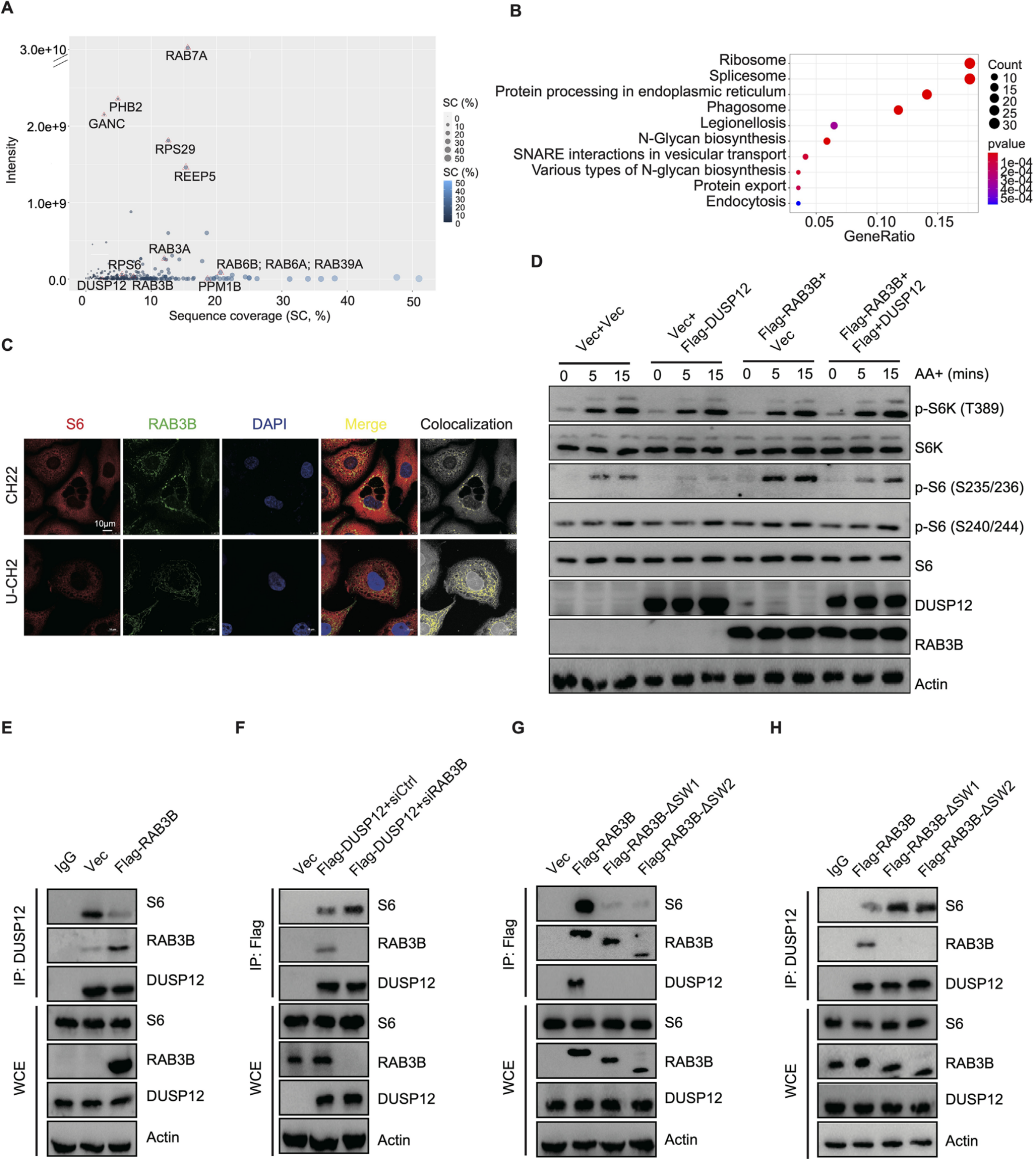
RAB3B interacts with DUSP12 and weakens its dephosphorylating of p‐S6. (A) Potential RAB3B‐regulated proteins identified by MS; (B) KEGG analysis of potential RAB3B‐regulated proteins identified by MS; (C) Immunofluorescence detection of S6 (red) and RAB3B (green) in CH22 and U‐CH2 cells. The rightmost graph shows the co‐localized signals between the red signal (S6) and the green signal (RAB3B). Scale bar, 10 µm; (D) WB analysis to assess the protein levels of mTORC1 signaling in CH22 cells with or without RAB3B and DUSP12 overexpression under AA stimulation; (E) Co‐IP assay to show the interaction between DUSP12 and S6 in CH22^Vec^ and CH22^Flag‐RAB3B^ cells; (F) Co‐IP assay to show the interaction between DUSP12 and S6 in CH22^Vec^ and CH22^Flag‐DUSP12^ cells with or without the treatment of siRAB3B; (G) Co‐IP assay to show the interaction between RAB3B and S6 in RAB3B overexpression and GTPase (ΔSW1 or ΔSW2) deletion CH22 cells; (H) Co‐IP assay to show the interaction between DUSP12 and S6 in RAB3B overexpression and GTPase (ΔSW1 or ΔSW2) deletion CH22 cells.

To further investigate the potential mechanism of RAB3B in mediating S6 phosphorylation (S235/236), mass spectrometry (MS) was used. In total, 322 potential RAB3B‐regulated proteins were identified, including RAB3A/B, RAB6A/B, RAB7A, etc (**Figure** **4**A; Data S2, Supporting Information). KEGG and GO analyses depicted that these proteins are enriched in the categories of ribosome, structural constituent of ribosome, protein targeting, and focal adhesion (Figure 4B; Figure S8A–C, Supporting Information). We shortlisted PI3K‐AKT‐mTOR signaling‐related proteins that were also identified as potential targets of RAB3B. As a result, ribosomal protein S6 was identified. Its relative intensity is shown in Figure S8D (Supporting Information). To validate our proteomic analysis, we further examined protein targets by co‐immunoprecipitation (IP) assay and successfully validated the binding between RAB3B and S6 (Figure S8E, Supporting Information). Via immunofluorescence, colocalization between S6 and RAB3B was also observed in chordoma cell lines, such as CH22 and U‐CH2 cells (Figure 4C).

Generally, S6K is an important protein kinase in phosphorylating S6. Although we did not identify the binding between RAB3B and S6K, our results revealed that knockdown of S6K1/2 via siRNA abandoned the RAB3B‐induced activation of S6 phosphorylation (Figure S8F,G, Supporting Information). In addition, to further investigate the roles of RAB3B in regulating S6K‐mediated S6 phosphorylation, we detected the weakened interaction between S6K and S6 in RAB3B knockdown CH22 cells, and vice versa (Figure S8H,I, Supporting Information).

As the kinase/substrate reaction is a “kiss‐and‐run” mechanism that may not require stable interactions between kinase and substrate, we further explored if phosphorylases regulated the RAB3B‐mediated S6 phosphorylation. Based on the results of MS, we found that RAB3B interacted with two phosphorylases (PPM1B and DUSP12; Figure 4A; Figure S9A,B, Supporting Information). DUSP12, but not PPM1B, dephosphorylated p‐S6 specially at the site of S235/236 under AA stimulation, whereas DUSP12 did not regulate AA starvation‐induced reduction of p‐S6 signal (Figure S9C–H, Supporting Information). In addition, the RAB3B‐mediated phosphorylation of S6 (S235/236) could be reversed by DUSP12 regulation (Figure 4D; Figure S9I, Supporting Information).

The physical binding between DUSP12 and RAB3B was identified, while inhibiting RAB3B enhanced the binding between DUSP12 and S6, and vice versa (Figure 4E,F; Figure S9J, Supporting Information). In addition, either deletion of switch I domain or switch II domain strongly reduced the interaction of RAB3B with S6 and DUSP12 (Figure 4G). Deletion of switch I domain or switch II domain alleviated the inhibitory effect of wide‐type RAB3B on the interaction of S6 and DUSP12 in vivo and in vitro binding assays (Figure 4H; Figure S9K, Supporting Information). Moreover, an in vitro dephosphorylation assay using eukaryotic protein expression and purification system revealed that RAB3B almost entirely reversed the DUSP12‐induced dephosphorylation of S6 at S235/236 (Figure S9L, Supporting Information).^[^ ^13^ ^]^ Thus, these results collectively suggest that the switch I and switch II domains of RAB3B weaken the interaction between DUSP12 and S6, thereby inhibiting the subsequent dephosphorylation of S6 by DUSP12.

In view of the tumorigenic roles of RAB3B in chordoma and above‐mentioned regulation of RAB3B/DUSP12 in mTORC1 signaling, we further explored the roles of DUSP12 in the stemness and tumorigenesis of chordoma. Unsurprisingly, our results revealed that deletion of DUSP12 increased the expression of stemness markers and promoted the proliferation of chordoma cells (Figure S10A–D, Supporting Information). Conversely, overexpression of DUSP12 reduced stemness marker expression and inhibited chordoma cell proliferation (Figure S10E–H, Supporting Information). These data further indicate the biological functions of RAB3B/DUSP12 in chordoma.

### 2.6 RAB3B Regulates the Cancer Stemness and Protein Translation Through S6

**Figure 5:**
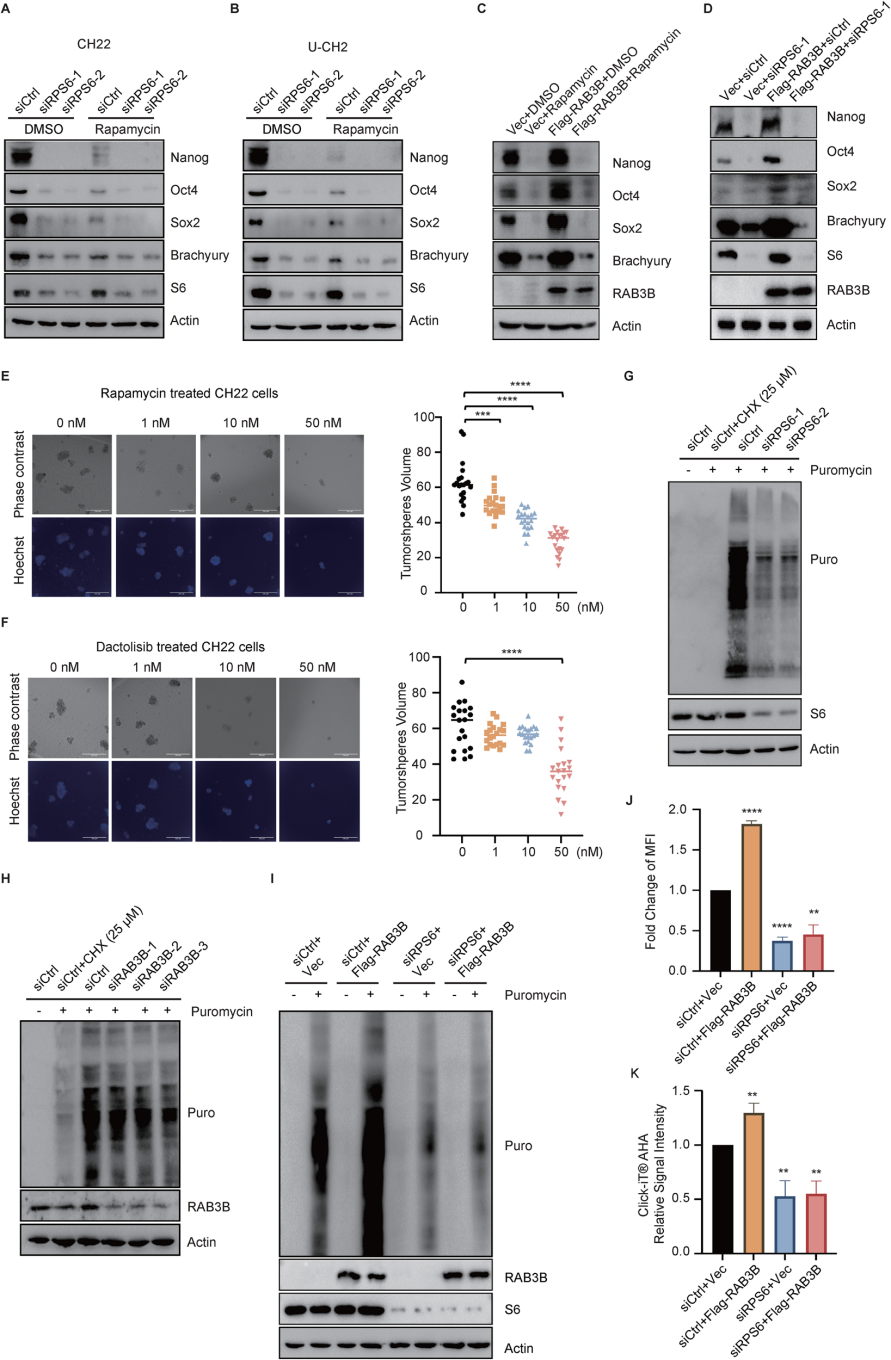
RAB3B regulates the cancer stemness and protein translation through S6. Protein levels of stemness markers in the CH22 (A) and U‐CH2 (B) cells with or without siRPS6 or rapamycin treatment; Protein levels of stemness markers in the CH22^Vec^ and CH22^Flag‐RAB3B^ cells with or without rapamycin (C) or siRPS6 (D) treatment; (E) Representative images of cultured CH22 tumorspheres after rapamycin treatment (left). Scale bar: 200 µm; Tumorspheres volume of CH22 after rapamycin treatment (right); (F) Representative images of cultured CH22 tumorspheres after dactolisib treatment (left). Scale bar: 200 µm; Tumorspheres volume of CH22 after dactolisib treatment (right); (G) The protein synthesis of CH22 cells after CHX or siRPS6 treatment in SUnSET assay; (H) The protein synthesis of CH22 cells with or without CHX or siRAB3B treatment in SUnSET assay; (I) The protein synthesis in CH22^Vec^ and CH22^Flag‐RAB3B^ cells with or without siRPS6 treatment in SUnSET assay; The protein synthesis in CH22^Vec^ and CH22^Flag‐RAB3B^ cells with or without siRPS6 treatment in the Click‐iT AHA Alexa Fluor 488 Protein Synthesis HCS Assay revealed by the flow cytometry (J) and fluorescence microscopy (K). **p < 0.01, ***p < 0.001, ****p < 0.0001.

As RAB3B regulates chordoma tumorigenicity and mTORC1/S6 signaling, we further explored whether it mediates the cancer stemness through S6. Knockdown of RPS6 via siRNA or inhibiting p‐S6 via rapamycin, a specific mTORC1 inhibitor, suppressed the expression levels of stemness markers, including Nanog, Oct4, Sox2, and Brachyury, in both CH22 and U‐CH2 cells (**Figure** **5**A,B). More importantly, reducing RPS6 expression levels or inhibiting its phosphorylation with rapamycin reversed the upregulation of stemness markers NANOG, OCT4, and SOX2 in RAB3B‐overexpressing cells (Figure 5C,D). To investigate the roles of targeting mTORC1 signaling on chordoma stemness, rapamycin and dactolisib (a dual ATP‐competitive PI3K and mTOR inhibitor) were used. We found both inhibitors diminishing the expression levels of RAB3B, along with stemness markers in CH22 and U‐CH2 cells (Figure S11A–F, Supporting Information). In the sphere formation assay, the sphere formation capacity of chordoma cells was markedly suppressed by rapamycin and dactolisib at a dose of 10 nm (Figure 5E,F; Figure S11G,H, Supporting Information).

As S6 plays an important role in protein translation, we investigated whether RAB3B could regulate protein translation through S6. Via the SUnSET assay, we measured protein synthesis in CH22 cells. Our results showed that either knockdown of RPS6 or RAB3B inhibits protein translation (Figure 5G,H). In addition, overexpression of RAB3B increased the protein translation (Figure S11I, Supporting Information), while knockdown of RPS6 blocked the upregulation of protein translation in RAB3B‐overexpressing cells (Figure 5I). To further validate the roles of RAB3B in S6‐associated protein translation, we used the Click‐iT AHA Alexa Fluor 488 Protein Synthesis HCS Assay. Based on the flow cytometry and fluorescence microscopy, the results also confirmed that RAB3B regulated protein translation via S6 (Figure 5J,K; Figure S11J,K, Supporting Information). Thus, these results indicate that RAB3B regulates the cancer stemness and protein translation through S6.

### 2.7 Targeting mTORC1 Signaling is a Potential Therapeutic Option for Chordoma

**Figure 6:**
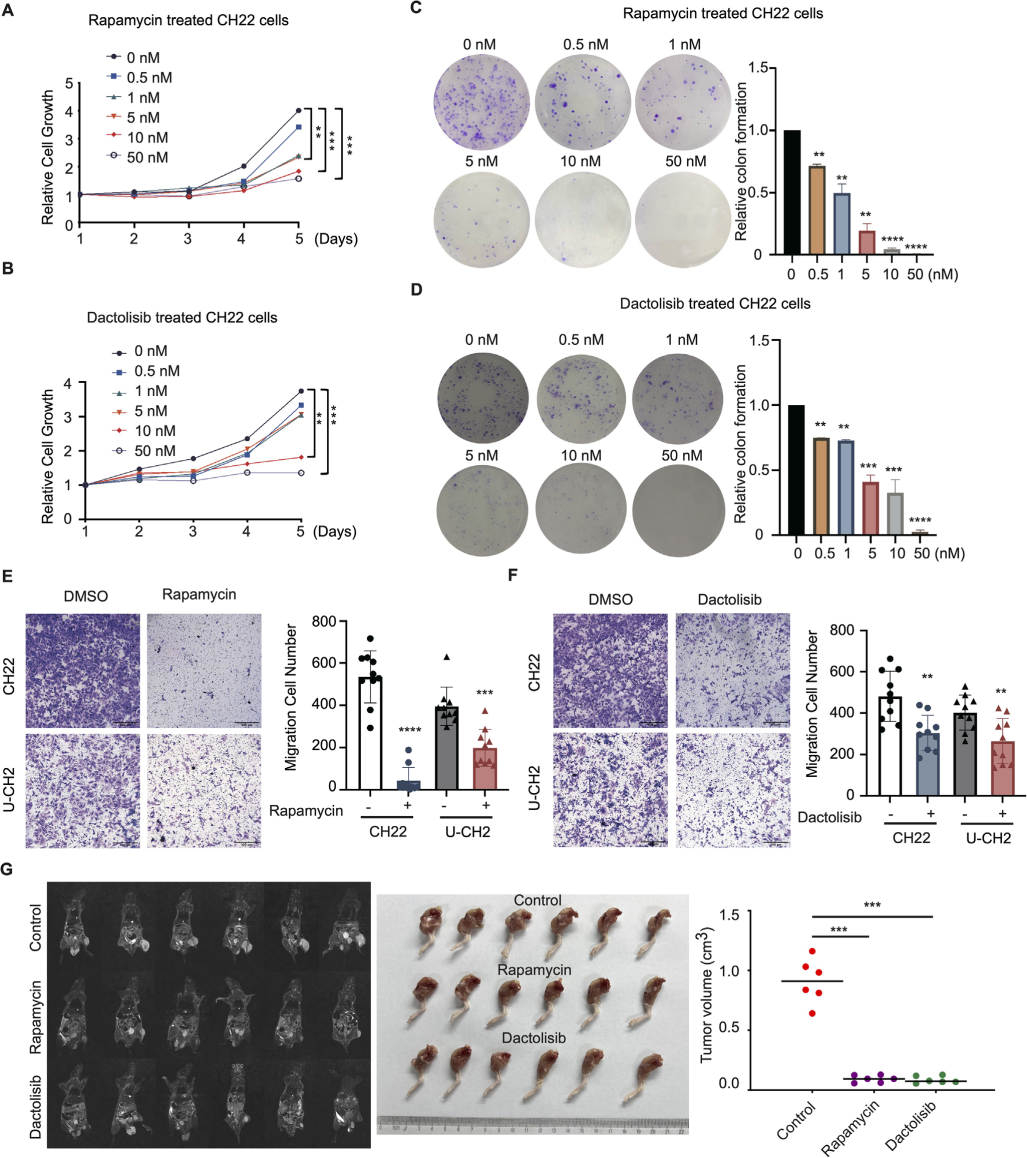
Targeting mTORC1 signaling is a potential therapeutic option for chordoma. MTT assay showed a significant inhibition of CH22 cell proliferation after rapamycin (A) and dactolisib (B) treatment in a time‐ and dose‐dependent manner. Colony formation of CH22 cells showed a significant inhibition of cell proliferation after rapamycin (C) and dactolisib (D) treatment in a time‐ and dose‐dependent manner: representative images of colony formation (left) and number of cell colony formation (right); Rapamycin (E) and dactolisib (F) inhibited the CH22 cell migration in transwell assay: representative images (left) and migration cell number (right); (G) Representative images of CH22‐bearing nude mice after dactolisib or rapamycin treatment (*n* = 6 for each group). MRI scans (left), tumor‐bearing legs (middle) and tumor volumes (right) of dactolisib‐, rapamycin‐ and PBS‐treated CH22‐bearing nude mice. **p < 0.01, ***p < 0.001, ****p < 0.0001.

Given the above‐mentioned crucial roles of RAB3B in regulating chordoma tumorigenicity and mTORC1/S6 signaling, we further explored the inhibitory effects of targeting mTORC1 signaling on chordoma via rapamycin and dactolisib. Rapamycin significantly weakened the cell proliferation of CH22 and U‐CH2 at the dose of 10 nm in a time‐dependent manner in MTT assay, while dactolisib achieved similar inhibitory effects on chordoma cell proliferation (**Figure** **6**A,B; Figure S12A,B, Supporting Information). As for colony formation assay, both rapamycin and dactolisib significantly suppressed the colonies at the concentration of 0.5 nm in CH22 and U‐CH2 cells (Figure 6C,D; Figure S12C,D, Supporting Information). Moreover, we also detected the effects of both inhibitors on chordoma cell migration. In the transwell assay, both could significantly weaken the migratory ability of CH22 and U‐CH2 cells (Figure 6E,F).

To gain further insight into therapeutic effects of these drugs on chordoma, we generated the CH22‐bearing nude mice. Rapamycin and dactolisib were found to dampen the chordoma tumorigenicity with significant decreases in TV (control: 0.9115 ± 0.1842 cm^3^; Rapamycin: 0.0941 ± 0.0303 cm^3^; Dactolisib: 0.0869 ± 0.0298 cm^3^; Figure 6G). The body weights of mice were not altered with either therapeutic modality (Figure S12E, Supporting Information). Taken together, both rapamycin and dactolisib diminished chordoma cell stemness, proliferation, migration, and tumorigenicity, which indicates the pivotal roles of mTORC1 signaling in chordoma tumorigenicity.

### 2.8 RAB3B/S6 Axis Indicates the Drug Susceptibility of mTORC1 Inhibitors for Chordoma

**Figure 7:**
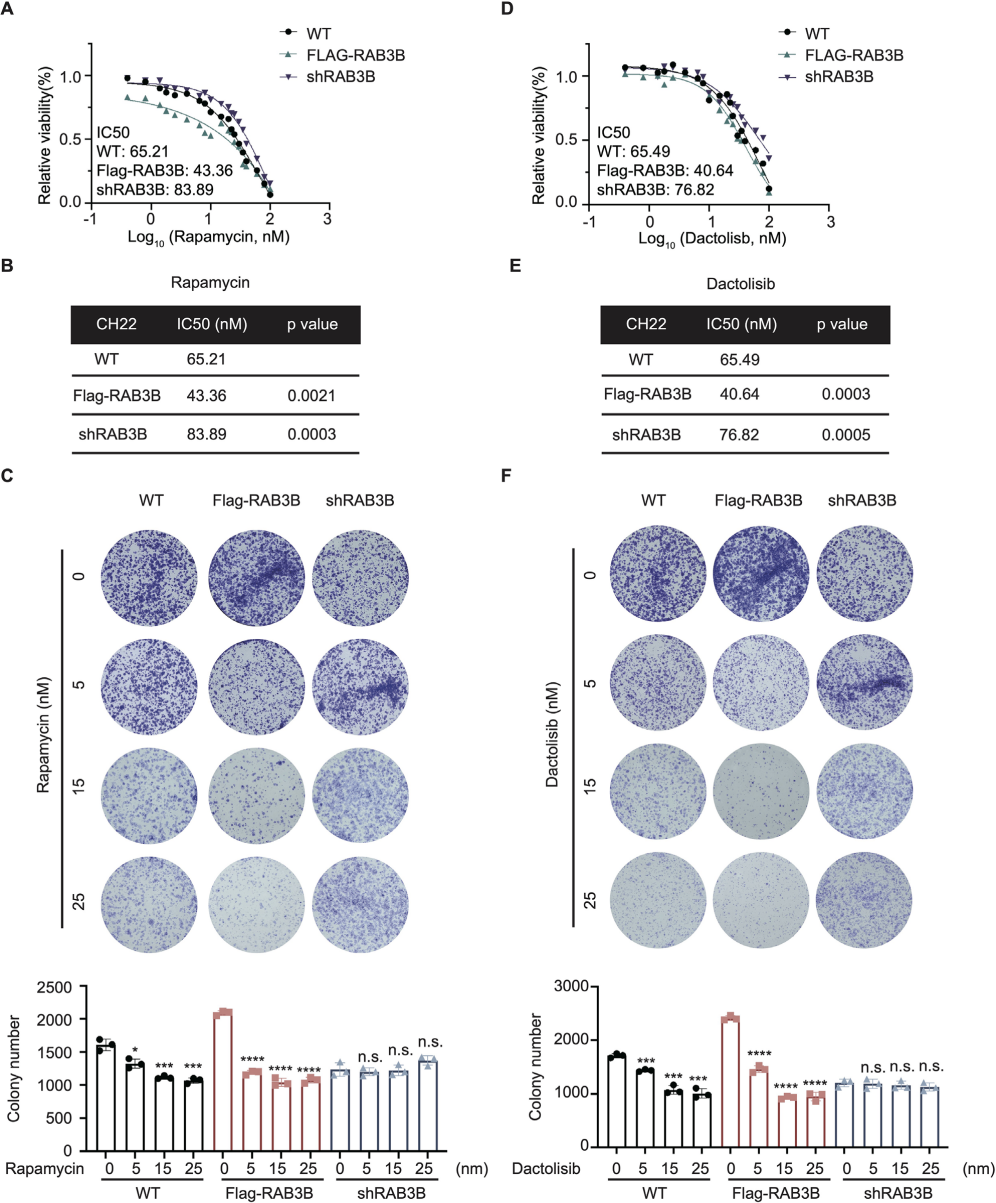
RAB3B/S6 axis indicates the drug susceptibility of mTORC1 inhibitors for chordoma. Relative viability (A) and IC_50_ values (B) of rapamycin for RAB3B wildtype, knockdown and overexpression CH22 cells; (C) Clone formation assay of rapamycin for RAB3B wildtype, knockdown and overexpression CH22 cells: Representative images of colony formation with or without treatment with rapamycin (up); number of cell colony formation (down); Relative viability (D) and IC_50_ values (E) of dactolisib rapamycin for RAB3B wildtype, knockdown and overexpression CH22 cells; (F) Clone formation assay of dactolisib for RAB3B wildtype, knockdown and overexpression CH22 cells: Representative images of colony formation with or without treatment with dactolisib (up); number of cell colony formation (down). ***p < 0.001, ****p < 0.0001.

mTORC1 inhibitors, such as PI‐103, AZD8055 and AZD2014, have been identified as potential therapeutic options.^[^ ^14^ ^]^ In addition, everolimus and sirolimus have also served as adjuvant therapies in clinical cases with progressive advanced chordoma.^[^ ^15^ ^]^ However, they only provide individual responses. In addition, no identified drug susceptibility indicator limits their individual application. As RAB3B‐induced S6 phosphorylation could be inhibited by dactolisib and rapamycin, we tested the predictive value of RAB3B in the dactolisib or rapamycin treatment for chordoma. Both dactolisib and rapamycin exhibited promising inhibitory potentials in CH22 cells and their therapeutic effects were correlated with RAB3B levels. The IC_50_ value of rapamycin in RAB3B wildtype CH22 cells was 65.21 nm (IC_50_ of RAB3B overexpression CH22 cells: 43.36 nm, *p* = 0.0021; IC_50_ of RAB3B knockdown CH22 cells: 83.89 nm, *p* = 0.0003; **Figure** **7**A,B). Rapamycin decreased the chordoma cell growth in CH22^Flag‐RAB3B^ and CH22^WT^, but not CH22^sgRAB3B^ (Figure 7C; Figure S13A,B, Supporting Information). As such, dactolisib also exhibited a similar trend. IC_50_ value of dactolisib was 65.49 nm in RAB3B wildtype CH22 cells, whereas that of RAB3B overexpression and knockdown CH22 cells was 40.64 nm (*p* = 0.0003) and 76.82 nm (*p* = 0.0005, Figure 7D,E). Based on clone formation and MTT assay, CH22 cells deficient in RAB3B were less sensitive to dactolisib, whereas high expression of RAB3B increased susceptibility to dactolisib (Figure 7F; Figure S13C,D, Supporting Information). As for other RAB3 isoforms, knockdown of RAB3A/C/D via siRNA did not regulate the drug susceptibility of mTORC1 inhibitors (Figure S13E, Supporting Information).

### 2.9 The Clinical Application of RAB3B/S6 Axis in Prognostic Prediction and Response to mTORC1‐Targeted Therapy in Chordoma Patients

We further sought to determine the predictive value of RAB3B/S6 axis in the prognostic evaluation and mTORC1 inhibitor response in chordoma patients. To this end, we compared the correlation between their protein levels and clinical outcomes. The protein levels of p‐S6 (S235/236) were also classified into high and low expression groups based on the median H‐score. The median H‐score of p‐S6 (S235/236) was 2.7 and high p‐S6 (S235/236) expression was defined as ≥ 2.7. As described in Figure S14A,B (Supporting Information), the expression of p‐S6 (S235/236) was significantly related to PFS (*p* = 1.557e–05) and OS (*p* = 1.667e–03) of chordoma patients.

**Figure 8:**
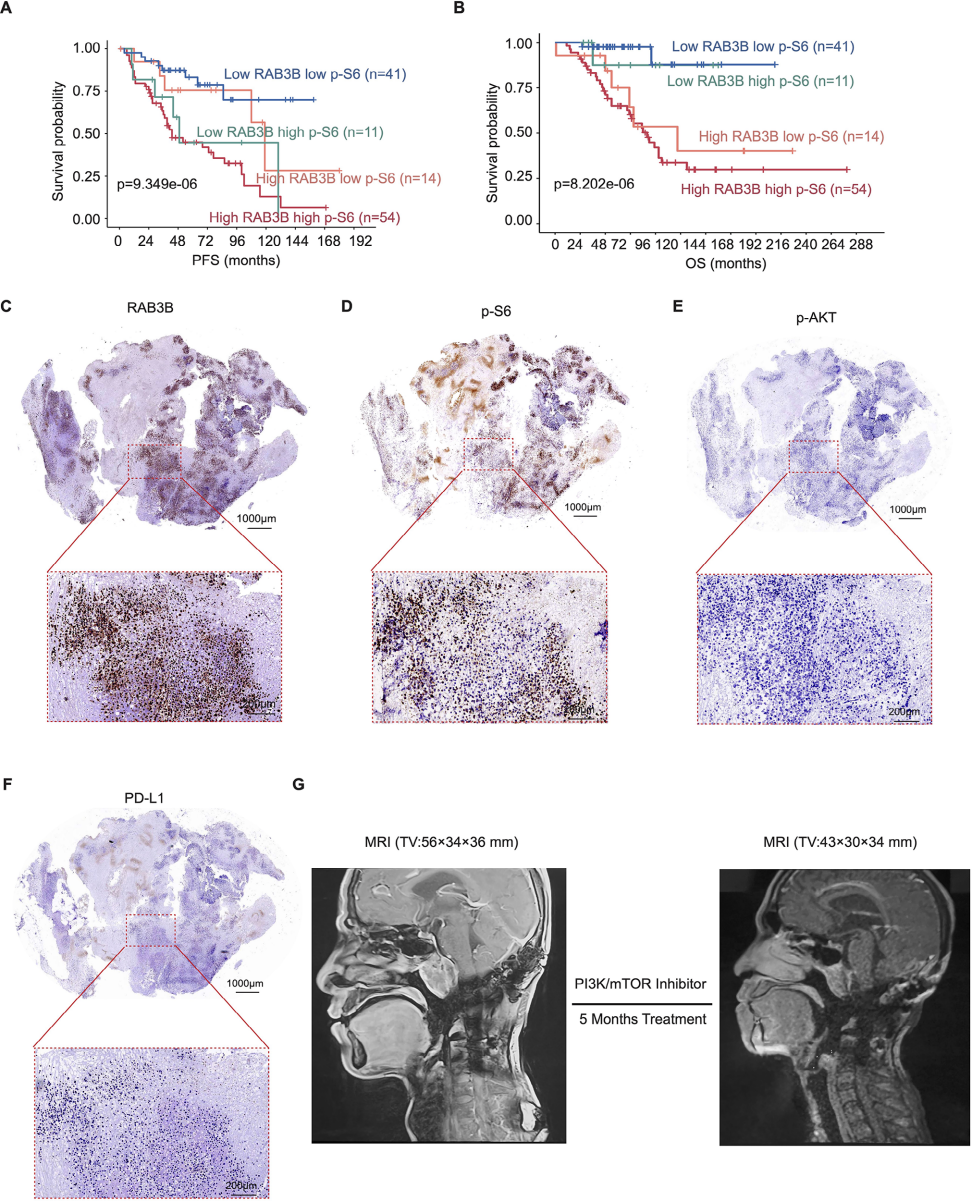
The clinical application of RAB3B/S6 axis in prognostic prediction and response to mTORC1‐targeted therapy in chordoma patients. Kaplan–Meier analysis of PFS (A) and OS (B) of chordoma patients in low and high RAB3B and p‐S6 (S235/236) group; The IHC of RAB3B (C), p‐S6 (S235/236) (D), p‐AKT (E) and PD‐L1 (F) in this advanced chordoma case before treatment. Scale bar, 200 and 1000 µm; (G) Representative MRI scans of this advanced chordoma case pre‐ and post‐treatment with PI3K/mTOR dual inhibitor.

To explore the combined effects of RAB3B and p‐S6 (S235/236), we investigated the patients’ survival among four groups on the basis of their levels. The representative IHC images in each group based on RAB3B and p‐S6 (S235/236) expression were presented in Figure S14C–F (Supporting Information). The results revealed that chordoma patients with high expression of RAB3B and p‐S6 (S235/236) had the poorest prognosis, while patients with low RAB3B and p‐S6 (S235/236) expression had the longest PFS (*p* = 9.349e–06, **Figure** **8**A) and OS (*p* = 8.202e–06, Figure 8B).

**Figure 9:**
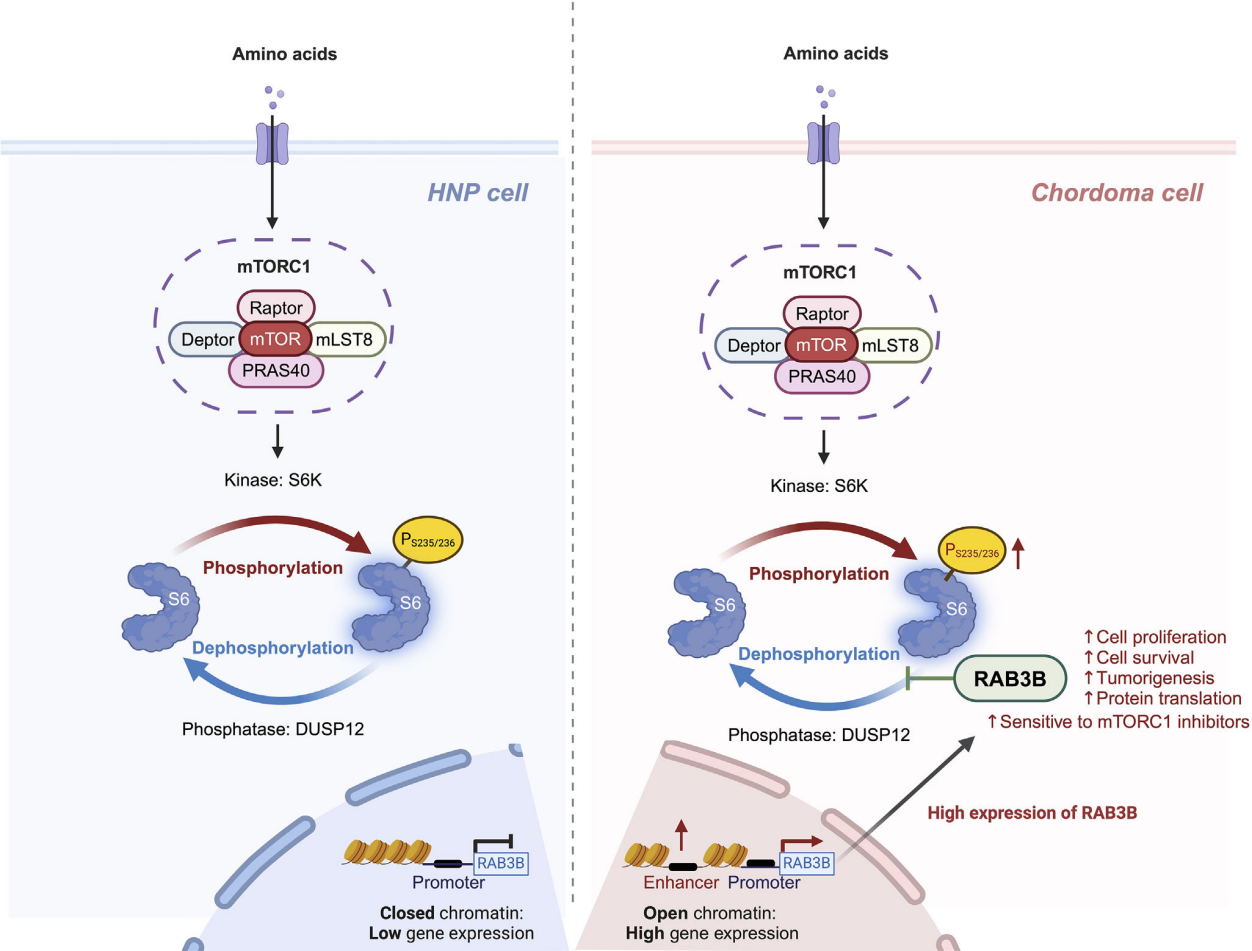
Schematic illustration of this study.

To further determine the role of RAB3B in predicting response to mTORC1 inhibitors in chordoma patients, we retrospectively collected advanced chordoma cases with the treatment of targeted therapies. As mTORC1 inhibitors were not commonly used in chordoma patients, only one patient with advanced clivus chordoma was identified. By testing the expression of key PI3K‐AKT‐mTOR markers (p‐AKT and p‐S6), common drug targets (EGFR, VEGFR, PDGFR, PD‐L1), immune cell markers (CD4, CD8) and RAB3B in this chordoma patient, we found high‐ and co‐expression of RAB3B and p‐S6 (S235/236), as well as EGFR (Figure 8C–F; Figure S14G–L, Supporting Information). After 5 months of treatment, the TV was significantly decreased in MRI scanning and symptom relief was obviously achieved (Figure 8G). The Response Evaluation Criteria in Solid Tumors Version 1.1 (RECIST 1.1) was used to evaluate the treatment outcome and partial response (PR) was achieved. Thus, high expression of RAB3B may serve as a potential indicator for the application of mTORC1 inhibitors in chordoma patients. Altogether, our data not only indicate a novel mechanism of RAB3B in regulating mTORC1 signaling by interacting with DUSP12 and blocking its dephosphorylation of p‐S6 at S235/236, but also provide a therapeutic strategy of mTORC1‐targeted therapy for advanced chordoma patients with aberrant RAB3B/p‐S6 hyperactivation (**Figure** **9**).

## 3 Discussion

Chordoma is derived from embryonic notochord with chemo‐/radio‐resistant nature and high relapse rate, thus its prognosis is relatively poor.^[^ ^3^, ^4^ ^]^ Nowadays, due to its rarity, the tumorigenic regulatory mechanisms remain elusive, which limits the identification of effective therapeutic targets and drug susceptibility indicators. In the current study, we identified RAB3B as a novel oncogenic regulator of chordoma, playing crucial roles in chordoma cell tumorigenicity and prognosis prediction. Mechanistically, we found that RAB3B could regulate mTORC1 signaling via working with S6 and DUSP12, a novel phosphorylase of S6, attenuating DUSP12‐mediated dephosphorylation of p‐S6 (S235/236). Based on functional experiments and clinical data, mTORC1‐targeted therapy could be regarded as a potential therapeutic modality for chordoma, especially with aberrant hyperactivation of RAB3B/p‐S6.

RABs constitute the largest family of small Ras‐like GTPases and serve as master regulators of intracellular membrane traffic.^[^ ^16^ ^]^ Generally, they control membrane identity and vesicle budding, motility, and fusion through recruiting effector proteins. As receptor signaling and trafficking participate in cancer biology, the aberrant expression of Rab GTPases often induces tumorigenesis.^[^ ^17^ ^]^ As a member of RAB GTPases, RAB3 isoforms (RAB3A/B/C/D) participate in the exocytotic fusion between membrane vesicles/granules and plasma membrane.^[^ ^18^ ^]^ Among the four RAB3 isoforms, RAB3D has been extensively studied and plays an oncogenic role in various tumor types, such as breast, colon, and brain tumors.^[^ ^19^ ^]^ RAB3A and RAB3C have been found to mediate the metastatic ability of hepatocellular carcinoma and colon cancers, indicating poor prognosis.^[^ ^20^ ^]^ However, tumorigenic roles of RAB3B remain obscure. In this study, we first identified its enhancer‐associated high transcription activity and prognostic value in chordoma, along with its tumorigenic and targeted potential.

Mechanistically, RAB3 isoform‐dependent activation of AKT/GSK3β pathway has been reported in several studies.^[^ ^19^ ^]^ In addition, RAB3C could modulate cancer cell migration through IL‐6‐JAK2‐STAT3 axis.^[^ ^20b^ ^]^ To investigate the tumorigenic mechanism of RAB3B in chordoma, RNA‐seq, and western blotting were applied and a strong correlation was found between RAB3B and PI3K‐AKT‐mTOR signaling, especially mTORC1 signaling. Among RAB GTPases, uncontrolled activation of RAB5 inhibited mTORC1 activity specific to AA stimulation;^[^ ^21^ ^]^ RAB1 has been identified as a tumorigenic regulator of colorectal cancer and AA promoted RAB1A GTP binding and interaction with mTORC1.^[^ ^22^ ^]^ In further identification of regulatory network between RAB3B and mTORC1 signaling, we found that RAB3B specifically stimulated the AA‐induced S6 phosphorylation at S235/236.

Ribosomal protein S6, a component of 40S ribosomal subunit, can be activated via its phosphorylation to control mRNA translation and protein synthesis in multiple physiopathologic activities.^[^ ^23^ ^]^ Generally, two classes of protein kinases have been reported to phosphorylate S6, that is p70 ribosomal protein S6 kinases (S6K1‐2) and p90 ribosomal S6 kinases (RSK1‐4), whereas the phosphorylase to dephosphorylate p‐S6 is less reported.^[^ ^24^ ^]^ Our data identified DUSP12 as a novel phosphorylase of p‐S6 (S235/236) and bound to RAB3B. Different from previous studies on the Ras oncogene family in regulating the phosphorylation of S6 via interaction with PI3K components, AKT‐S473 phosphorylation and ERK/RSK axis,^[^ ^24^, ^25^ ^]^ we identified a novel regulatory mechanism of S6 phosphorylation by Ras family member RAB3B that RAB3B interacted with S6 and DUSP12, weakening DUSP12‐mediated dephosphorylation of p‐S6 (S235/236).

Due to the oncogenic roles of mTORC1 signaling, its inhibitors, such as rapamycin and sirolimus, have been applied in the clinical treatment of various tumor types.^[^ ^26^ ^]^ Dactolisib, also called BEZ235, is a dual PI3K‐mTOR inhibitor. In a phase 2a randomized, placebo‐controlled clinical trial, dactolisib plus RAD001 selectively inhibited TORC1 downstream of mTOR, with good (decrease in the rate of infections) and safe response.^[^ ^27^ ^]^ Many clinical trials have been performed to investigate its effects in the treatment of malignancies, including advanced renal cell carcinoma (NCT01453595), metastatic or unresectable malignant PEComa (NCT01690871), advanced solid tumors (NCT01343498), and advanced breast cancer (NCT00620594).

Nowadays, targeting mTORC1 signaling have served as an adjuvant therapy and provided clinical benefits to a certain extent for some chordoma patients.^[^ ^5^, ^10^, ^15^ ^]^ However, their regulatory mechanisms and sensitivity markers have not been reported in chordoma. In this study, we found that RAB3B‐mediated mTORC1 signaling is tightly associated with chordoma cell proliferation, migration, and tumorigenicity, which may explain the clinical application of mTORC1‐targeted therapy in chordoma patients. Moreover, based on in vitro experiment and clinical chordoma case, RAB3B was identified as a predictor for response to mTORC1‐targeted therapy in chordoma.

Although our results revealed that mTORC1‐targeted therapy may serve as a potential option for chordoma with abnormal RAB3B/p‐S6 hyperactivation, the immunosuppressive properties remain a potential obstacle to the clinical application of mTORC1 inhibition in cancer therapy. Based on previous studies, in many human tumors including tuberous sclerosis complex (TSC) and lymphangioleiomyomatosis (LAM), mTORC1‐hyperactivity upregulates B7‐H3/CD276 to inhibit antitumor T cells and drive tumor immune evasion, while mTORC1 inhibitors could display strong antitumor effects and increase antitumor immunity.^[^ ^28^ ^]^ In line with this, our results showed that the chordoma case with aberrant RAB3B/p‐S6 hyperactivation exhibited very low expression of CD4^+^ and CD8^+^ T cells and mTORC1 inhibition benefited this patient. However, we are unable to assess the immunosuppressive effects of mTORC1 inhibition in this study, due to no murine cell lines or autochthonous tumor models available for chordoma. Thus, we cannot completely rule out the potential immunosuppressive role of mTORC1 inhibition in treating chordoma patients.

## 4 Conclusion

This study uncovers RAB3B as the novel regulator of mTORC1 pathway in mediating site‐specific S6 phosphorylation (S235/236) via physically binding to S6 and DUSP12, concomitantly inhibiting DUSP12‐mediated dephosphorylation of p‐S6. Our findings also highlight that RAB3B overexpression drives oncogenesis of chordoma and renders chordoma patients prone to mTORC1‐targeted therapy.

## 5 Experimental Section

### Cell Lines and Culture

The human chordoma cell line CH22 was obtained from the Chordoma Foundation;^[^ ^29^ ^]^ U‐CH2 and MUG‐Chor1 were purchased from the American Type Culture Collection (ATCC); and human HEK293T was purchased from the Type Culture Collection of the Chinese Academy of Sciences (Shanghai, China). The culture condition is described as cultured in Supporting Information.

### Data Collection, Processing, and Analysis of DEGs

The RNA‐seq data of 3 NP samples (control group), along with formalin‐fixed paraffin‐embedded (FFPE) tissues of 7 spine chordoma and 7 NP samples were obtained from Shanghai General Hospital. The RNA sequencing (RNA‐seq) data of chordoma from the spine were downloaded from Sequence Read Archive (SRX2935165) including 30 chordoma samples (https://www.ncbi.nlm.nih.gov/sra/).^[^ ^30^ ^]^ The RNA‐seq data pertaining to the downstream signaling of RAB3B came from RAB3B wildtype and knockdown CH22 cells. Chordoma cell line sequencing data is available at https://www.chordomafoundation.org and chordoma tissues sequencing data were uncovered by the team reported in the previous study.^[^ ^11^ ^]^ External validation of chordoma and notochord gene expression profiling were found in Chordoma Project Supporting Information (http://www.xavierlab2.mgh.harvard.edu/chordoma/index.html). The detailed methods for RNA sequencing are described in Supporting Information.

The FFPE samples were dewaxed and digested using pressure cycling technology (PCT). After desalting and tandem mass tags (TMT) labeling, fractionation was performed with a Waters XBridge Peptide BEH C18 column under a DIONEX UltiMate 3000 Liquid Chromatogram. Then, liquid chromatography‐mass spectrometry/ mass spectrometry (LC‐MS/MS) was used to identify the candidate protein expression in different tissues. The detailed methods were described in Supporting Information.

### TMA and Immunohistochemistry (IHC) Analysis

The TMA of chordoma samples were obtained from Shanghai General Hospital, Shanghai Tenth People's Hospital, and the First Affiliated Hospital of Zhengzhou University including 120 samples from 75 patients. The control groups were the paraffin embedded embryonic NP (30 samples) from the First Affiliated Hospital of Zhengzhou University. The chordoma TMA and control tissue sections were immunohistochemically stained using a kit from Dako (Copenhagen). Antibodies were presented in Table S1 (Supporting Information). Nuclei were counterstained with hematoxylin (Vector Laboratories). Immunostaining on each slide was evaluated by two experienced pathologists with H‐score. H‐score = Σpi(i + 1) where i represents the intensity score and pi represents the percentage of cells with that intensity.

### Quantitative Real‐Time PCR (qRT‐PCR)

Based on the manufacturer's instructions, total RNA was extracted using TRIzol reagent (Invitrogen). Then column purification and reverse transcription were performed using PrimeScript 1st Strand cDNA Synthesis Kit (Takara). All qRT‐PCR analyses were performed using Fast SYBR Green (Takara). Primers were presented in Table S2 (Supporting Information).

### Knockdown and Overexpression Cell Line Generation

HEK293T cells were transfected using pLVX‐empty vector, pLVX‐Flag‐RAB3B, pLVX‐Flag‐PPM1B or pLVX‐Flag‐DUSP12 and shNC or shRAB3B and sgNC or sgRAB3B, and Flag‐RAB3B‐Rescue, and sgNC, sgPPM1B or sgDUSP12, and sgEnhancer (F) or sgEnhancer (R), together with psPAX2 and pMD2.G, adding PEI in a ratio of 1:2. Viral supernatant was collected and used to infect CH22 cells. After 48 h of infection and subsequent culturing, puromycin was administered to successfully select infected cells. Detailed primers of plasmid construction were described in Supporting Information.

### Establishment of Truncated Cell Lines

To construct the truncated cell lines, specific truncation mutant plasmids were designed by deleting predefined domains (SWITCH I or SWITCH II of RAB3B, see schematic diagram). The truncated sequences were then inserted into the pLVX vector. All constructs were verified by Sanger sequencing to confirm the correct insertion and frame integrity. For stable expression, HEK293T cells were transfected using sgNC or sgRAB3B, together with psPAX2 and pMD2.G, adding PEI in a ratio of 1:2. Viral supernatant was collected and used to infect CH22 cells. After 48 h of infection and subsequent culturing, puromycin was administered to successfully select infected cells. After selection and validation, repeat the above procedure to establish truncated cell lines using truncation mutant plasmids. The expression of truncated proteins was validated by Western blotting.

### Knockdown of RAB3B, S6K1, and S6K2 by siRNA Transfection

The human RAB3B, rpS6, S6K1, and S6K2 siRNA were synthesized by GenePharma and RAB3B, rpS6, S6K1, or S6K2 knockdown was performed in human chordoma cells by siRNA transfection. A total of 2 × 10^5^ chordoma cells per well were seeded in 12‐well plates. The synthetic human RAB3B, rpS6, S6K1, or S6K2 siRNA (20 nmol L^−1^) or nonspecific siRNA (20 nmol L^−1^) were transfected using Lipofectamine RNAiMax Reagent (Invitrogen). The targeting sequences are described in Table S3 (Supporting Information).

### AA Starvation and Restimulation

For AA stimulation, cells were treated as previously described.^[^ ^31^ ^]^ In brief, cells were incubated in AA‐free indicated medium for 60 min and then stimulated with AAs for indicated time periods.

### IP and Western Blotting

IP and western blotting were performed as previously described.^[^ ^32^ ^]^ Transfected cells were lysed in lysis buffer (150 mm NaCl, 0.5% NP‐40, 1 mm EDTA, 10% glycerophosphate, 50 mm Tris‐Cl, pH 7.4, and a cocktail of protease inhibitors). After 30 min, cell lysates were isolated via centrifugation for 15 min (12 000 rpm, 4 °C). For IP, cleared cell lysates were incubated with M2 beads (Sigma) or HA‐conjugated beads (Abmart) (4 °C, 120 min). For samples of MS, beads were stored in liquid nitrogen after cleaned and drained. The detailed methods are described in Supporting Information. To detect endogenous protein interactions, cell lysates were incubated with 50 µl of protein A/G agarose beads (Beyotime Biotechnology) and 6 µl of indicated antibody (4 °C, overnight). After extensive washing, beads were boiled and resolved via SDS‐polyacrylamide gel electrophoresis (SDS‐PAGE), then analyzed by immunoblotting. The proteins were detected using the Odyssey system (LI‐COR Biosciences, Lincoln, USA).

### Glutathione S‐Transferase (GST)‐Pull Down

To determine whether the RAB3B protein and its truncated forms regulate the binding between DUSP12 and RPS6, RAB3B protein with MBP‐tag, DUSP12 protein with a GST‐tag and RPS6 protein with a His‐tag, were purchased from IPODIX Biotech. For the GST pull‐down assay, purified GST‐tagged magnetic beads were first washed 2–3 times with binding buffer to remove storage impurities and resuspended. Purified GST‐tag protein was then added to the beads and incubated at 4 °C with slow agitation for 60 min to allow specific binding. After washes with washing buffer, cell lysate containing the prey protein was added and incubated at 4 °C with gentle agitation overnight. The beads were washed again to remove unbound proteins. After extensive washing, beads were boiled and resolved via SDS‐PAGE, then analyzed by immunoblotting.

### Immunofluorescence Staining

The cells were washed three times with phosphate‐buffered saline (PBS), fixed for 15 min with 4% paraformaldehyde and permeabilized with 0.2% Triton X‐100 for 20 min on ice. Nonspecific binding was then blocked by incubation for 30 min with 5% Bovine Serum Albumin (BSA) in PBS and cells were incubated for 2 h with specific primary antibodies (indicated in figures). After washing three times, cells were incubated for another 1 h with secondary antibodies (Alexa Fluor 488‐conjugated anti‐mouse IgG (A21202), Alexa Fluor 488‐conjugated anti‐rabbit IgG (A21206) or Alexa Fluor 555‐conjugated anti‐mouse IgG (A31570) (Invitrogen). Nuclei were counterstained with DAPI (Sigma–Aldrich). All images were collected with a confocal microscope (Zeiss LSM 780).

### Cell Proliferation Assay

Cells were exposed to various concentrations of rapamycin (Selleckchem)/dactolisib (Selleckchem) and RAB3B/nonspecific siRNA for respective time points as indicated. Cell proliferation ability was assessed by MTT assays. Chordoma cells were seeded in a 96‐well plate (3 × 10^3^ cells per well for CH22 and U‐CH2). Cells were harvested every 24 h, and MTT (Sigma–Aldrich) was added for 4 h. The reactions were stopped by adding dimethyl sulfoxide (DMSO) solution for 20 min, and samples were measured at 490 nm.

### Cellular Viability Assay

RAB3B wildtype, knockdown, and overexpression CH22 cells were seeded in a 96‐well plate (2000 cells per well) and treated with different concentrations of rapamycin/dactolisib from 0 to 200 nm for 48 h. Cellular viability levels were quantified using CellTiter‐Glo (Promega, G7573) on multi‐plate reader (TECAN). Relative viability evaluated by cellular ATP was normalized to the corresponding untreated condition. Non‐linear regression models were fitted by Graphpad (version 8.4.3) and IC_50_ was obtained.

### Clone Formation Assay

Chordoma cells were seeded in a six‐well plate (500 cells per well) after transfection with RAB3B/nonspecific siRNA or different concentrations of rapamycin/dactolisib for 48 h. RAB3B wildtype, knockdown, and overexpression CH22 cells were seeded in a six‐well plate (2000 cells per well) with or without rapamycin/dactolisib. Cells were cultured for 2 weeks, fixed with 4% paraformaldehyde, stained with crystal violet (Sigma–Aldrich), and rinsed with PBS three times. The stains were dissolved in 95% ethanol and diluted tenfold for 10 min. Then the samples were measured at 570 nm and counted.

### Tumor Sphere Formation Assay

Chordoma cells were seeded at 2000 cells per well into 96‐well ultra‐low attachment plates (Corning) and cultured in DMEM/F12 (Sigma–Aldrich) supplemented with 50x B27 Supplement (Invitrogen), Basic Fibroblast Growth Factor (Peprotech), Epidermal Growth Factor (Sigma–Aldrich), Insulin (Invitrogen) and Bovine Serum Albumin (Sigma–Aldrich) after transfection with RAB3B/nonspecific siRNA for 48 h or treatment with different concentrations of rapamycin/dactolisib. They were cultured for 2 weeks and recorded using Cytation5 (BioTek), counted after staining with Hoechst 33342 (Beyotime Biotechnology).

### Transwell Assay

CH22^WT^, CH22^shRAB3B^ cells, and chordoma cells treated with rapamycin/dactolisib (25 nm) were cultured in low serum (5% FBS) medium and seeded in upper chamber of transwell 24‐well plates (Corning) with 8 µm pore filters. The lower chamber was added with complete medium within 10% FBS. After 12 h, cells were fixed with 4% paraformaldehyde, stained with crystal violet (Sigma‐Aldrich), and rinsed with PBS three times. The migration was photographed under Cytation 5 (BioTek).

### Wound Healing Assay

Cells were seeded in a six‐well plate (5 × 10^5^ cells per well) and treated with Mitomycin C (Sigma–Aldrich, 1 µg mL^−1^) for 1 h when incubated to reach confluence. The cells were scratched with a pipette tip and washed with phosphate‐buffered saline to remove detached cells. Chordoma cells were cultured in complete medium with 1% FBS treated with PBS or rapamycin/dactolisib (25 nm). Cells were photographed at 0, 6, 12, 24, and 48 h. The wound was calculated as follows: Relative percentage of wound healing (%) = (A0 – A1)/A0 × 100, A0: initial wound area, A1: remaining area of wound at the metering point.

### Surface Sensing of Translation (SUnSET)

Chordoma cells were seeded at 25 000 cells per well into six‐well plate (Corning) and cultured in RPMI‐1640 culture medium one day before the experiment. On the day of the experiment, replace the medium with fresh medium. Next, expose cells to 5 µg mL^−1^ puromycin directly diluted in the media for 15 min at 37 °C and 5% CO_2_, and remove media and rinse cells twice with cold PBS. Replace the medium with fresh medium again and incubated for 1 h at 37 °C and 5% CO_2_. Negative controls were pretreated with 25 µm cycloheximide (CHX) before experiments. For AA stimulation, Chordoma cells were incubated in AA‐free indicated medium for 60 min and then stimulated with AAs for 60 min before *SUnSET*. Cells were then lysed with RIPA buffer containing protease and phosphatase inhibitors, and centrifuged to collect the protein‐containing supernatant. Equal amounts of protein were loaded onto an SDS‐PAGE gel, separated, and analyzed by immunoblotting. The membrane was blocked, incubated with an anti‐puromycin antibody overnight, followed by an HRP‐conjugated secondary antibody, and the signal was detected using an enhanced chemiluminescence reagent.

### Click‐iT AHA Alexa Fluor 488 Protein Synthesis HCS Assay

CH22 cells were cultured in methionine‐free 1640 supplemented with essential nutrients. Cells were incubated with 50 µm Click‐iT AHA for 30 min. After incubation, cells were fixed with 3.7% formaldehyde in PBS for 15 min, washed with 3% BSA in PBS, and permeabilized using 0.5% Triton X‐100 in PBS for 20 min. Nascent protein synthesis was detected using Click chemistry with Alexa Fluor 488 alkyne, followed by Hoechst 33342 nuclear staining. Fluorescence imaging was performed using the Cytation 5 (BioTek) platform, with signal quantification in the FITC and DAPI channels. Data were analyzed and statistical methods were applied to assess significance.

### Eukaryotic Protein Expression and Purification

HEK293T cells were seeded at a density of 1.5 × 10⁶ cells per 100 mm dish and transfected with 5 µg of Flag‐RPS6, Flag‐DUSP12 or Flag‐RAB3B plasmid using Lipofectamine 2000, following the manufacturer's protocol. After 36–48 h of incubation at 37 °C with 5% CO₂, cells were harvested using a cell scraper in 1 ml of ice‐cold 1× PBS, collected by centrifugation at 1000 × g for 1 min at 4 °C, and lysed in 500 µl of IP lysis buffer on ice for 30 min with occasional mixing. The lysate was clarified by centrifugation at 12 000 × g for 15 min at 4 °C, and the supernatant was transferred to a new tube. For immunoprecipitation, M2 beads were added to HEK293T cell lysate and incubated with gentle rotation at 4 °C for 1 h. Beads were collected by centrifugation at 1000 × g for 1 min at 4 °C, washed three times with 0.5 mL of ice‐cold IP lysis buffer. Add Flag‐peptide in IP lysis buffer, and incubated with gentle rotation at 4 °C for 1 h. lysis was collected by centrifugation at 1000 × g for 1 min at 4 °C, protein concentration was determined using a BCA kit, western blot was used to detect the expression of purified protein and phosphorylation.

### In Vitro Dephosphorylation Assay

The dephosphorylation assay was performed by incubating 0.5 µg of RPS6 and with or without 1 µg of DUSP12 and with or without 1 µg of RAB3B in 50 µl of phosphatase buffer at 4 °C for 60min and 37 °C for 30 min. The reaction was stopped by adding 1 bead volume of 2× SDS loading buffer, followed by heating at 100 °C for 10 min. Half of each sample was subjected to SDS‐PAGE, and phosphatase activity was assessed by Coomassie blue staining. The remaining half was used to detect the phosphorylation status of S6 at S235/236 via Western blotting. Following phosphorylation detection, the membrane was stripped with 0.8 N NaOH for 30 s, and total S6 protein substrate was detected using an anti‐S6 antibody.

### Tumor Xenografts

To evaluate therapeutic effects of targeting mTORC1 signaling, a total of 5 × 10^6^ CH22 cells in 50 µL of RPMI‐1640 were injected into the tibias of male nude mice (5–6 weeks of age, 18–20 g, Shanghai SLAC Laboratory Animal Co., Ltd, China). These mice were randomized and categorized into three groups: PBS (*n* = 6), rapamycin (*n* = 6) and dactolisib (*n* = 6). CH22‐bearing mice were treated daily with rapamycin (3.5 mg kg^−1^ day^−1^),^[^ ^33^ ^]^ dactolisib (40 mg kg^−1^ day^−1^),^[^ ^34^ ^]^ or PBS orally on day 8 after tumor inoculation for 3 weeks. To determine the roles of RAB3B in chordoma tumorigenicity, RAB3B wildtype, and knockout CH22 cells were injected according to the above‐mentioned method. Tumor size was assessed by micro‐MRI under general anesthesia and analyzed with ITK‐SNAP 3.6 software by two independent investigators. The formula V = ab^2^/2 was used to calculate tumor volumes (V represents volume; a represents length; b represents width). Ultimately, mice were anesthetized and sacrificed by cervical dislocation.

### Study Approval

This study was approved by the institutional review board of Shanghai General Hospital (approval number: 2021SQ013). The written informed consent for tissue and clinical data collection was signed by all patients or their legal guardians. The animal experiment was administered based on the guidelines of the Institutional Animal Institutional Animal Care and Use Committees with approval number of 2023AWS032.

### Statistical Analysis

All the quantification analysis was performed using SPSS Statistics (version 26.0). Experimental data were presented as the mean ± SD and repeated at least three times. The figures were drawn by GraphPad Prism 9 software and R Version 3.6.1. The Kaplan–Meier survival curve was generated to assess the survival rate. Statistical significance was determined by the Mann‐Whitney U test, unpaired two‐sided Weich's *t*‐test, and two‐tailed Student's *t*‐test, as indicated in figure legends. The reported *p* values were adjusted to account for multiple comparisons. Significance was indicated by *p* < 0.05 and denoted in figures by *p < 0.05, **p < 0.01, ***p < 0.001, or ****p < 0.0001.

## Conflict of Interest

The authors declare no conflict of interest.

## Author Contributions

J.G., J.J., R.H., and S.W. contributed equally to this work, and all should be considered as first author. T.M. conceived the project. D.W.S., J.R.X., P.W., and T.M. designed experiments; J.X.G., J.L.J., S.H.S., Y.Z., Y.A.L., and T.M. performed experiments. R.Z.H. and S.Q.W. performed bioinformatics analysis. T.M., D.W.S., H.B.Y., W.S., and Z.Q.H. obtained the tumor tissues. J.L. and Z.Y.C. performed pathological analysis. D.W.S., J.R.X., P.W., and T.M. interpreted and analyzed data; and J.X.G., R.Z.H., and T.M. wrote the manuscript with comments from all authors.

## Supporting information



Supporting Information

Supplemental Data 1

Supplemental Data 2

## Data Availability

Sequencing data of ATAC‐seq for BMSC and CH22 cells, along with the RNA‐seq of CH22 cells, and primary chordoma samples are deposited at the Sequence Read Archive (SRA) database (PRJNA738543). The ATAC‐seq and ChIP‐seq of H3K27ac data are available at NCBI's GEO database under the accession number GSE109794. Chordoma cell line sequencing data are available at https://www.chordomafoundation.org.
