# Supplementary material for: RAB3B Dictates mTORC1/S6 Signaling in Chordoma and Predicts Response to mTORC1‐Targeted Therapy
Source: Adv Sci (Weinh). 2025 Mar 26;12(19):2415384. doi: 10.1002/advs.202415384 (PMC12097036; doi:10.1002/advs.202415384)
Supplement: Supplementary file 1 — Supporting Information [file ADVS-12-2415384-s003.pdf]

ADVANCED SCIENCE
Open Access

Supporting Information

for *Adv. Sci.*, DOI 10.1002/advs.202415384

RAB3B Dictates mTORC1/S6 Signaling in Chordoma and Predicts Response to mTORC1-Targeted Therapy

Jianxuan Gao, Jiali Jin, Runzhi Huang, Siqiao Wang, Sihui Song, Yu Zhang, Yongai Li, Jun Lin, Zhengyan Chang, Zongqiang Huang, Wei Sun, Huabin Yin, Dianwen Song, Jianru Xiao*, Ping Wang* and Tong Meng**

Supporting Information

RAB3B Dictates mTORC1/rpS6 Signaling in Chordoma and Predicts Response to mTORC1 Inhibitors

Jianxuan Gao†, Jiali Jin†, Runzhi Huang†, Siqiao Wang†, Sihui Song, Yu Zhang, Yongai Li, Jun Lin, Zhengyan Chang, Zongqiang Huang, Wei Sun, Huabin Yin, Dianwen Song, Jianru Xiao*, Ping Wang*, Tong Meng**

Supplementary Methods

Cell culture condition

CH22 was cultured in RPMI-1640 medium (Invitrogen); U-CH2 and MUG-Chor1 were maintained in Iscove's modified Dulbecco's medium (IMDM; Invitrogen): RPMI-1640 medium (4:1); and HEK293T was cultured in Dulbecco's Modified Eagle Medium (DMEM; Invitrogen), supplemented with 10% fetal bovine serum (FBS; Gibco) and 1% penicillin/ streptomycin (PS; Invitrogen). The U-CH2 and MUG-Chor1 were also supplemented with 1% l-glutamine (Invitrogen).

RNA sequencing

*Library preparation:*Upon confirmation of sample quality, 1-3 ug of total RNA from each sample were utilized as starting material for the construction of transcriptome sequencing libraries. According to the instructions of VAHTS Universal V6 RNA-seq Library Prep Kit for Illumina® (NR604-01/02), libraries were constructed using different index labels. For the qualified Total RNA samples, eukaryotic mRNA was enriched using poly-T oligo-attached magnetic beads. Subsequently, fragmentation buffer was added to break the mRNA into short fragments. Using mRNA as a template, single-strand cDNA was synthesized with random hexamers. Then, buffer, dNTPs, RNase H and DNA polymerase I were added to generate double-stranded cDNA. double-stranded cDNA was purified using AMPure P beads or QiaQuick PCR purification kit. The purified double stranded cDNA underwent end repair, adding a tailing and connecting sequencing connector, then the fragment size was selected, PCR enrichment was conducted to obtain the final cDNA library.

Library examination： After library construction completed, initial quantification was first conducted using Qubit® RNA Assay Kit in Qubit® 3.0, followed by diluting the library to 1ng/ul. The library's insert size was assessed using the Agilent Bioanalyzer 2100 system (Agilent Technologies, CA, USA).

Once the insert size met the expected criteria, the library's effective concentration (10 nm) was precisely quantified using the Bio-RAD CFX 96 fluorescence quantitative PCR instrument, the reagent used was Bio-RAD KIT iQ SYBR GRN.

Library clustering and sequencing: Clustering and sequencing were performed on the NovaSeq 6000 S4 platform, using the NovaSeq 6000 S4 Reagent kit V1.5. The paired-end sequencing program (PE) was executed, yielding 150bp paired-end sequencing reads.

Quantitative proteomics analyses

Dewaxing treatment: Place the staining rack with FFPE samples into the oven at 37°C for 30 min. Then transfer the staining rack into a staining jar, add heptane to the staining jar where the sample is submerged, cover the jar lid, stand at room temperature for 10 min, discard the liquid, and repeat once. Add 100% ethanol into the staining jar to cover the sample, stand at room temperature for 5 min, and discard the liquid. Then add 90% ethanol and 75% ethanol in sequence to repeat this treatment. Adding deionized water into the dyeing jar to soak the sample; take out samples in turn and transfer into PCT tubes with a blade. Add 12.5 ul pH=10 100 mM tris-HCl(Tris hydrochloride) to the PCT tubes containing the samples, and place them on a horizontal shaker at 600 rpm for 30 min at 95°C.

PCT based protein digestion: Triethylammonium bicarbonate (TEAB), Tris (2-carboxyethyl) phosphine (TCEP) and iodoacetamide (IAA) were added for reduction and alkylation under 45000 psi, with 30 s high pressure and 10 s ambient pressure per cycle (30 °C for 90 cycles). TEAB was then added into PCT tube to decrease the concentration of Urea to lower than 1.5 M. Then Trypsin and Lys-Cwere added for protein digestion under 20000 psi, with 50 s high pressure and 10 s ambient pressure per cycle (30 °C for 120 cycles). Tryptic peptides were transferred into 1.5 mL tubes and digestion was then terminated by 15 μL 10% TFA.

Desalting and TMT labeling: pH of samples was between 2 and 3. SOLAμ (Thermo Fisher Scientific™, San Jose, USA) was applied for desalting and TMTpro 16plex Isobaric Label Reagent Set was applied for TMT labeling according to their user guide.

High pH Fractionation: Fractionation was performed with a Waters XBridge Peptide BEH C18 column (300 Å, 5 μm × 4.6 mm × 250 mm) under a DIONEX UltiMate 3000 Liquid Chromatogram. Mobile phase A was 10 mM ammonium hydroxide (pH=10), and mobile phase B was 98% ACN, 10 mM ammonium hydroxide (pH=10). Peptides were collected every one minute from 5% ACN to 35% ACN with a flowrate of 0.5 ml/min in 60 min, and then combined into 30 fractions. After SpeedVac dried, the

30 fraction samples were resuspended with 2% ACN, 0.1% Formic Acid and then sent for LC-MS analysis.

MS analysis: LC-MS/MS with the nanoflow DIONEX UltiMate 3000 RSLCnano System coupled to an Orbitrap Exploris 480 mass spectrometer (Thermo Scientific™, San Jose, USA), which equipped with a FAIMS Pro™ (Thermo Scientific™, San Jose, USA), in data dependent acquisition (DDA) mode. The analytical column (1.9 µm, 120 Å, 150 mm*75 µm i.d.) was used. The m/z range of MS1 was 375-1800 with the resolution at 60,000, normalized AGC target of 300% with the intensity threshold of 2e4, and maximum ion injection time (max IT) of 50 ms. MS/MS experiment were performed with a resolution at 30000, normalized AGC target of 200%, and max IT of 86 ms. isolation window was set to 0.7 m/z and first mass was set to 110 m/z. The fasta file was Human SwissProt_20200715_20368_IRT_con.fasta (download from https://www.uniprot.org/uniprot/). MS data was performed using Proteome Discoverer (Version 2.5.0.400, Thermo Scientific™, San Jose, USA) earch engine against the human protein database downloaded from SwissProt (version 15/07/2020; 20368 proteins), with a precursor ion mass tolerance of 10 ppm and fragment ion mass tolerance of 0.02 Da. Briefly, TMT pro-plex labels to lysine and N-terminus, and carbamidomethylation of cysteine were set as static modifications. A cut-off criterion of a q-value of 0.01, corresponding to a 1% false-discovery rate (FDR) was set for the filtered of identified peptides with highly confident peptide hits.

DNA Gel Electrophoresis: Genomic DNA was extracted from CH22 infected with sgRAB3B-Enhancer. DNA concentration and purity were measured with a NanoDrop spectrophotometer. PCR amplification was performed using Bio-rad with primer RAB3B-Enhancer (1) and RAB3B-Enhancer (2). A 1% agarose gel was prepared by dissolving 1 g of agarose (Tsingke Biotech) in 100 mL of 1x TAE buffer (40 mM Tris-acetate, 1 mM EDTA, pH 8.0), heated until dissolved, cooled to 60°C, and mixed with ethidium bromide (0.5 µg/mL). The solution was poured into a gel tray with a comb and solidified at room temperature. DNA samples were combined with 10x loading dye (Tsingke Biotech). A 5000 bp DNA ladder (Tsingke Biotech) was prepared as a molecular weight marker. After the gel solidified, it was placed in an electrophoresis tank with 1x TAE buffer. DNA samples and the DNA ladder were loaded into the wells. Post-electrophoresis, the gel was visualized using a UV transilluminator (Bio-Rad) and photographed with a GelDoc XR+ imaging system (Bio-Rad).

Supplementary Table

Table S1. Antibodies used in this study.

Product name	Company	Product code
Anti-S6K1 antibody	Abcam	ab32529
Anti-S6K1 (phospho T389) antibody	Abcam	ab126818
S6 Ribosomal Protein Rabbit mAb	Cell Signaling Technology	#2217
S6 Ribosomal Protein (54D2) Mouse mAb	Cell Signaling Technology	#2317
Phospho-S6 Ribosomal Protein (Ser235/236) Rabbit mAb	Cell Signaling Technology	#4858
Phospho-S6 Ribosomal Protein (Ser240/244) Rabbit mAb	Cell Signaling Technology	#5364
p70 S6 Kinase Antibody	Cell Signaling Technology	#9202
Phospho-p70 S6 Kinase (Thr389) (108D2) Rabbit mAb	Cell Signaling Technology	#9234
Anti-RSK1 p90 antibody	Abcam	ab32114
Anti-RSK1 (phospho S380) p90 antibody	Abcam	ab32203
Phoshpo-p90RSK (Thr359/Ser363) Antibody	Cell Signaling Technology	#9344
RSK2 Recombinant Rabbit Monoclonal Antibody	HUABIO	JE63-19
RSK3 Polyclonal antibody	Proteintech	14446-1-A
PKAα/β/γ cat Rabbit Polyclonal Antibody	HUABIO	ER64618
Anti-PKA alpha/beta/gamma (catalytic subunit) (phospho T197) Antibody	Abcam	Ab75991
DUSP12 Monoclonal Antibody	Proteintech	67101-1-Ig
DUSP12 Polyclonal antibody	Proteintech	15667-1-AP
PPM1B Polyclonal antibody	Proteintech	13193-1-AP
Akt Antibody	Cell Signaling Technology	#9272
Phospho-Akt (Ser473) Rabbit mAb	Cell Signaling Technology	#4060
Phospho-Akt (Thr308) (D25E6) XP Rabbit mAb	Cell Signaling Technology	#13038
p44/42 MAPK (Erk1/2) Antibody	Cell Signaling Technology	#9102

Phospho-p44/42 MAPK (ERK1/2) (Thr202/Tyr204) (D13.14.4E) XP Rabbit mAb	Cell Signaling Technology	#4370
Anti-mTOR (phospho S2448) antibody	Abcam	ab109268
mTOR Rabbit mAb	Cell Signaling Technology	#2983
Phospho-4E-BP1 (Thr37/46) Rabbit mAb	Cell Signaling Technology	#2855
Anti-Nanog antibody	Abcam	ab109250
NANOG Rabbit pAb	ABclonal Technology	A14150
SOX2 (D1C7J) XP Rabbit mAb	Cell Signaling Technology	#14962
Anti-OCT4 antibody	Abcam	ab19857
Anti-Brachyury/Bry antibody	Abcam	ab209665
RAB3B/C Polyclonal Antibody	Proteintech	15774-1-AP
RAB3B monoclonal antibody (M02), clone 1A7	Abnova	H00005865-M02
Anti-beta Tubulin antibody	Hangzhou Bio Technology	db3285
Anti-Puromycin Antibody, clone 12D10	Sigma-Aldrich	MABE343

Table S2. Primer sequences used in this study.

Gene	Primer	
human RAB3B	Forward 5’- -3’	CCGCTATGCTGATGACACGTT
	Reverse 5’- -3’	ACGGTAGACTGTCTTCACCTTG
human TBXT	Forward 5’- -3’	TATGAGCCTCGAATCCACATAGT
	Reverse 5’- -3’	CCTCGTTCTGATAAGCAGTCAC
human NANOG	Forward 5’- -3’	AAGGTCCCGGTCAAGAAACAG
	Reverse 5’- -3’	CTTCTGCGTCACACCATTGC
human OCT4	Forward 5’- -3’	CTTGAATCCCGAATGGAAAGGG
	Reverse 5’- -3’	GTGTATATCCCAGGGTGATCCTC
human SOX2	Forward 5’- -3’	TACAGCATGTCCTACTCGCAG
	Reverse 5’- -3’	GAGGAAGAGGTAACCACAGGG
human Actin	Forward 5’- -3’	CATGTACGTTGCTATCCAGGC

	Reverse 5’- -3’	CTCCTTAATGTCACGCACGAT
RAB3B-Enhancer(1)	Forward 5’- -3’	GTCTTAAGTAATCCACCAGCCT
	Reverse 5’- -3’	CAAAACAAAACACCTCCAGCTT
RAB3B-Enhancer(2)	Forward 5’- -3’	TTGGCAATTCATATACACAGTT
	Reverse 5’- -3’	GTTTAGTGTGTCCAACATAGG
RAB3B-Enhancer-Validation	Forward 5’- -3’	TCACCCAGGTTGGAAGTGCA
	Reverse 5’- -3’	TCTTTAAAAAAGGATGTATC

Table S3. Synthetic siRNA, shRNA and Flag-Tagged RNA in this study.

Gene	Sequence	
	Sense (5’-3’)	Antisense (5’-3’)
RAB3B (1)	GGGCUUCAUUCUGAUGUAUTT	AUACAUCAGAAUGAAGCCCTT
RAB3B (2)	CAGCUUGGGUUUGAUUUCUTT	AGAAAUCAAACCCAAGCUGTT
RAB3B (3)	GACCAGACCAUCCCGCAGCTT	GCUGCGGGAUGGUCUGGUCTT
RPS6(1)	CGAGCUUCUACUUCUAAGUTT	ACUUAGAAGUAGAAGCUCGTT
RPS6(2)	CCGCCAGUAUGUUGUAAGATT	UCUUACAACAUACUGGCGGTT
S6K1	CCGGAGAAUAUCAUGCUUATT	UAAGCAUGAUAUUCUCCGGTT
S6K2	GGCCGUGUUUGAUUUGGAUTT	AUCCAAAUCAAACACGGCCTT
DUSP12 (1)	GUCGAAGUGUGGCCAUAAUTT	AUUAUGGCCACACUUCGACTT
DUSP12 (2)	GCAGAAUUUACCUCAAGAATT	UUCUUGAGGUAAAUUCUGCTT
shRAB3B	CCGGGATGAGTTGCTGCTATTCTTTCTCGAG AAAGAATAGCAGCAACTCATCTTTTTG	AATTCAAAAAGATGAGTTGCTGCTATTCTTT CTCGAGAAAGAATAGCAGCAACTCATC

sgRAB3B	CACCGGTAGTCAAAATTCTGGTCAG	AAACCTGACCAGAATTTTGACTACC
Flag-RAB3B	CCGCTCGAGATGGCTTCAGTGACAGA	CGCGGATCCCTAGCATGAGCAGTTCT
Flag-RAB3B-Rescue	GCATCTGATCAGAATTTTGATTACATGTTTAAACT	GTAATCAAAATTCTGATCAGATGCATCTTTGACTC
sgPPM1B (1)	CACCGCCAGCAAGACCTCCCCCAGG	AAACCCTGGGGGAGGTCTTGCTGGC
sgPPM1B (2)	CACCGAATCCACATAGAGAAAGTGA	AAACTCACTTTCTCTATGTGGATTC
sgDUSP12(1)	CACCGCATCACGGCCGTGCTAACAG	AAACCTGTTAGCACGGCCGTGATGC
sgDUSP12(2)	CACCGGCTCCAGATTCTCAAACCAG	AAACCTGGTTTGAGAATCTGGAGCC
Flag-PPM1B	ATGGGTGCATTTTTGGATAAAC	TCATATTTTTCACCACTCATC
Flag-DUSP12	ATGTTGGAGGCTCCGGGCCCGA	TCATATTTTTCCTGTTTGTGAT
sgEnhancer (F)	CACCGTGGCCTCCCAAAGTGTTGGG	AAACCCCAACACTTTGGGAGGCCAC
sgEnhancer (R)	CACCGATGTCTTCCGGGTGATAGGC	AAACGCCTATCACCCGGAAGACATC
Switch I	TTCCGCTATGCTGATGTGGGCATCGACTTCAAGGT	GAAGTCGATGCCCACATCAGCATAGCGGAAGAGGA
Switch II	AAACTGCAGATCTGGCGTGGGGCCATGGGCTTCAT	GCCCATGGCCCCACGCCAGATCTGCAGTTTCACCC

Table S4 H-score of RAB3B in chordoma and NP

H-score	RAB3B

	Chordoma	NP
Number	120	30
Mean	4.26	2.18
SD	0.64	0.36
p	<0.000	

H-score, Histochemistry score; SD, standard deviation; NP, nucleus pulposus

Supplementary Figure

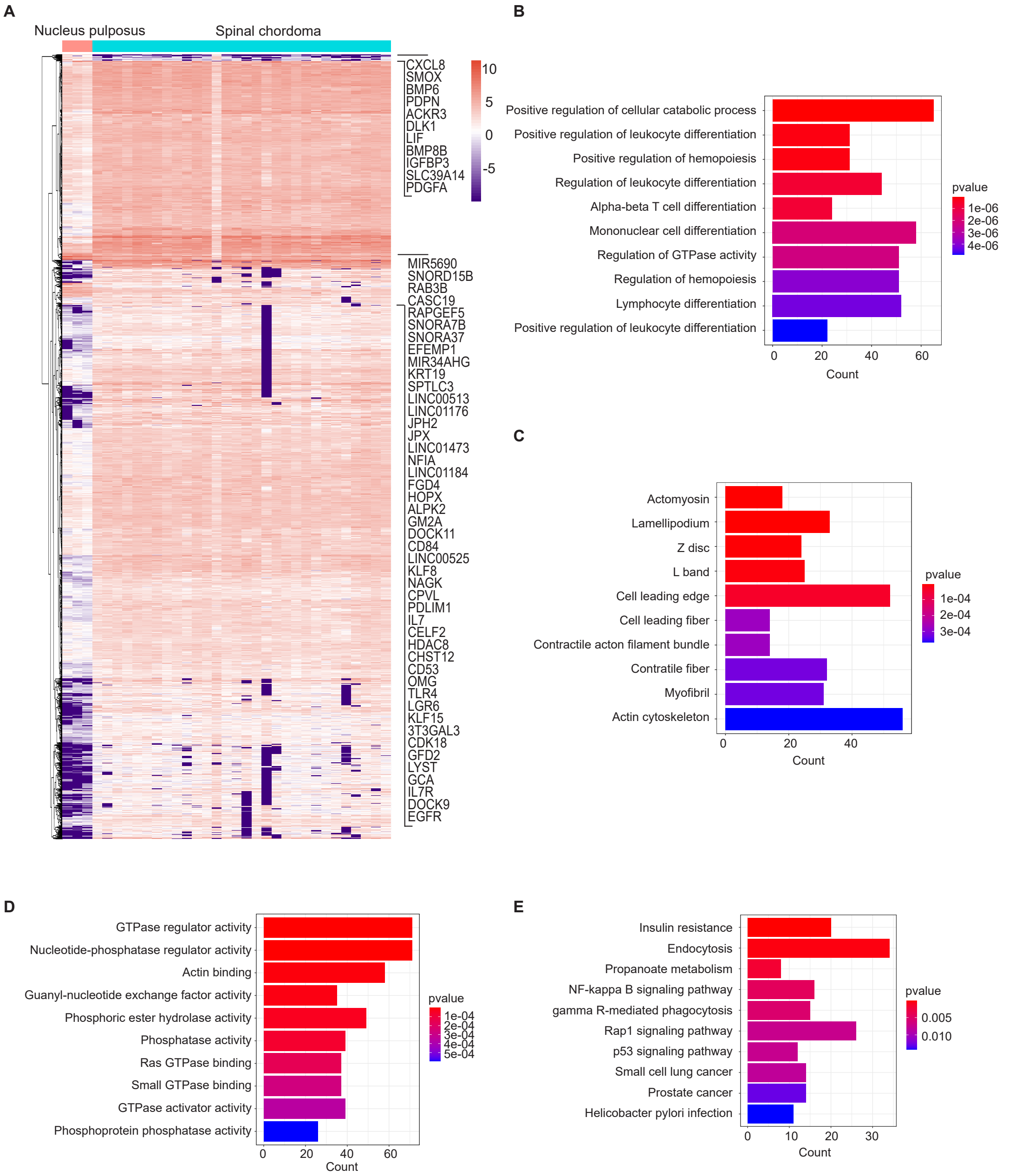

Figure S1. Identification of the potential tumorigenic genes in chordoma

(A) Heatmap of DEGs between chordoma (n=30) and NP (n=3) samples; BP (B), CC (C) and MF (D) module of GO analysis of DEGs between chordoma and NP; (E) KEGG analysis of DEGs between chordoma and NP.

Abbreviations: DEGs, differentially expressed genes; NP, Nucleus pulposus; BP, Biological process; CC, Cellular component; MF, Molecular function; GO, Gene Ontology; KEGG, Kyoto Encyclopedia of Genes and Genomes.

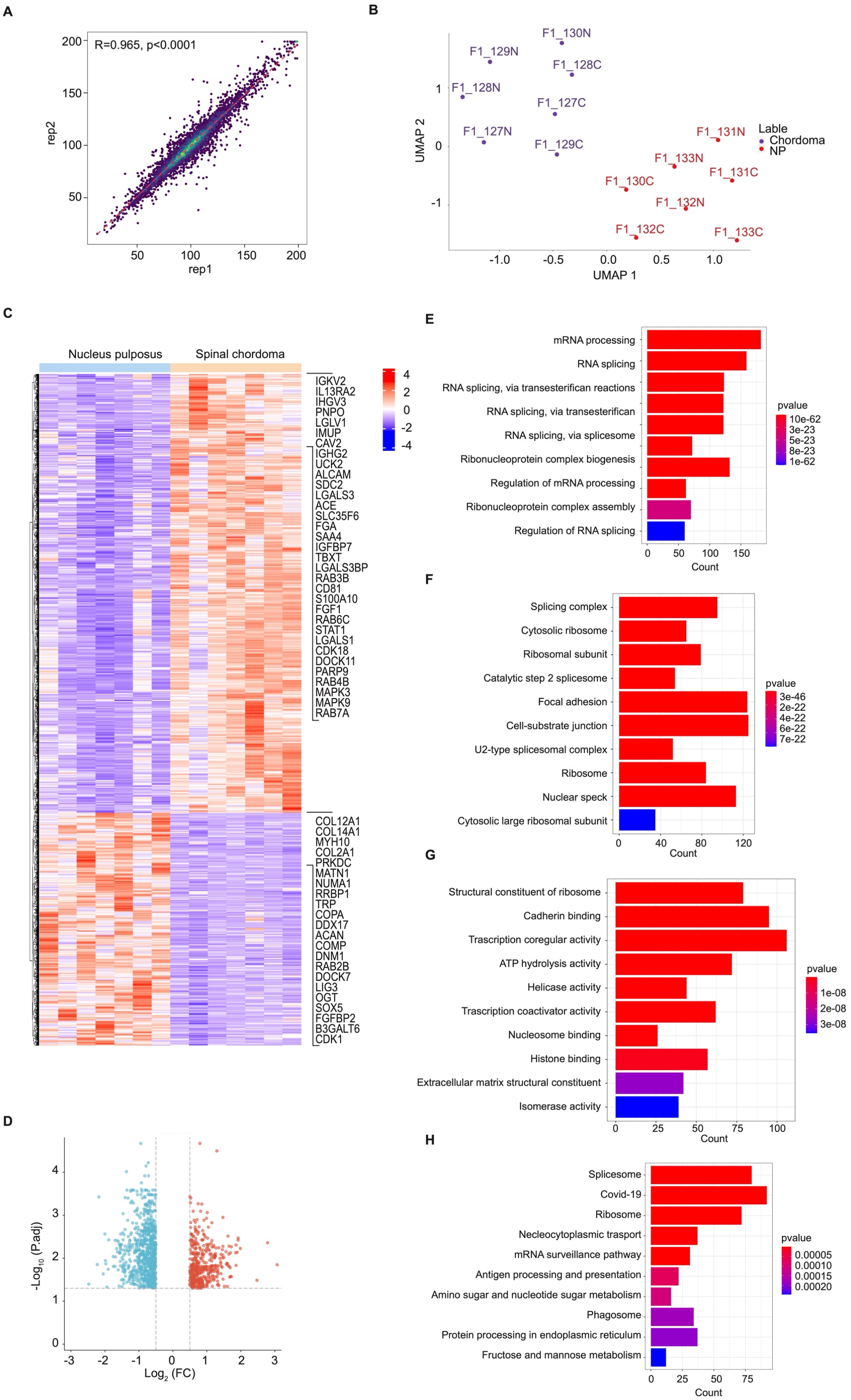

A
R=0.965, p<0.0001
rep2
rep1
200
150
100
50
B
F1_130N
F1_129N
F1_128C
F1_128N
F1_127C
F1_127N
F1_129C
F1_131N
F1_133N
F1_131C
F1_130C
F1_132N
F1_132C
F1_133C
Lable
Chordoma
NP
UMAP 2
UMAP 1
-1.0
-0.5
0.0
0.5
1.0
C
Nucleus pulposus
Spinal chordoma
IGKV2
IL13RA2
IHGV3
PNPO
LGLV1
IMUP
CAV2
IGHG2
UCK2
ALCAM
SDC2
LGALS3
ACE
SLC35F6
FGA
SAA4
IGFBP7
TBXT
LGALS3BP
RAB3B
CD81
S100A10
FGF1
RAB6C
STAT1
LGALS1
CDK18
DOCK11
PARP9
RAB4B
MAPK3
MAPK9
RAB7A
COL12A1
COL14A1
MYH10
COL2A1
PRKDC
MATN1
NUMA1
RRBP1
TRP
COPA
DDX17
ACAN
COMP
DNM1
RAB2B
DOCK7
LIG3
OGT
SOX5
FGFBP2
B3GALT6
CDK1
4
2
0
-2
-4
D
-Log10 (P.adj)
Log2 (FC)
-3
-2
-1
0
1
2
3
E
mRNA processing
RNA splicing
RNA splicing, via transesterifican reactions
RNA splicing, via transesterifican
RNA splicing, via splicesome
Ribonucleoprotein complex biogenesis
Regulation of mRNA processing
Ribonucleoprotein complex assembly
Regulation of RNA splicing
pvalue
10e-62
3e-23
5e-23
8e-23
1e-62
Count
F
Splicing complex
Cytosolic ribosome
Ribosomal subunit
Catalytic step 2 splicesome
Focal adhesion
Cell-substrate junction
U2-type splicesomal complex
Ribosome
Nuclear speck
Cytosolic large ribosomal subunit
pvalue
3e-46
2e-22
4e-22
6e-22
7e-22
Count
G
Structural constituent of ribosome
Cadherin binding
Trascription coregular activity
ATP hydrolysis activity
Helicase activity
Trascription coactivator activity
Nucleosome binding
Histone binding
Extracellular matrix structural constituent
Isomerase activity
pvalue
1e-08
2e-08
3e-08
Count
H
Splicesome
Covid-19
Ribosome
Necleocytoplasmic trasport
mRNA surveillance pathway
Antigen processing and presentation
Amino sugar and nucleotide sugar metabolism
Phagosome
Protein processing in endoplasmic reticulum
Fructose and mannose metabolism
pvalue
0.00005
0.00010
0.00015
0.00020
Count

Figure S2. Identification of the potential tumorigenic proteins in chordoma

(A) Scatter plot of correlation coefficients between one chordoma sample and its replicate sample; (B) UMAP of chordoma and NP samples in the proteomics analysis; (C) Heatmap of DEPs between chordoma (n=7) and NP (n=7) samples; (D) The volcano plot of DEPs; BP (E), CC (F) and MF (G) module of GO analysis of differentially expressed proteins between chordoma and NP; (H) KEGG analysis of DEPs between chordoma and NP.

Abbreviations: NP, Nucleus pulposus; DEPs, differentially expressed proteins; BP, Biological process; CC, Cellular component; MF, Molecular function; GO, Gene Ontology; KEGG, Kyoto Encyclopedia of Genes and Genomes.

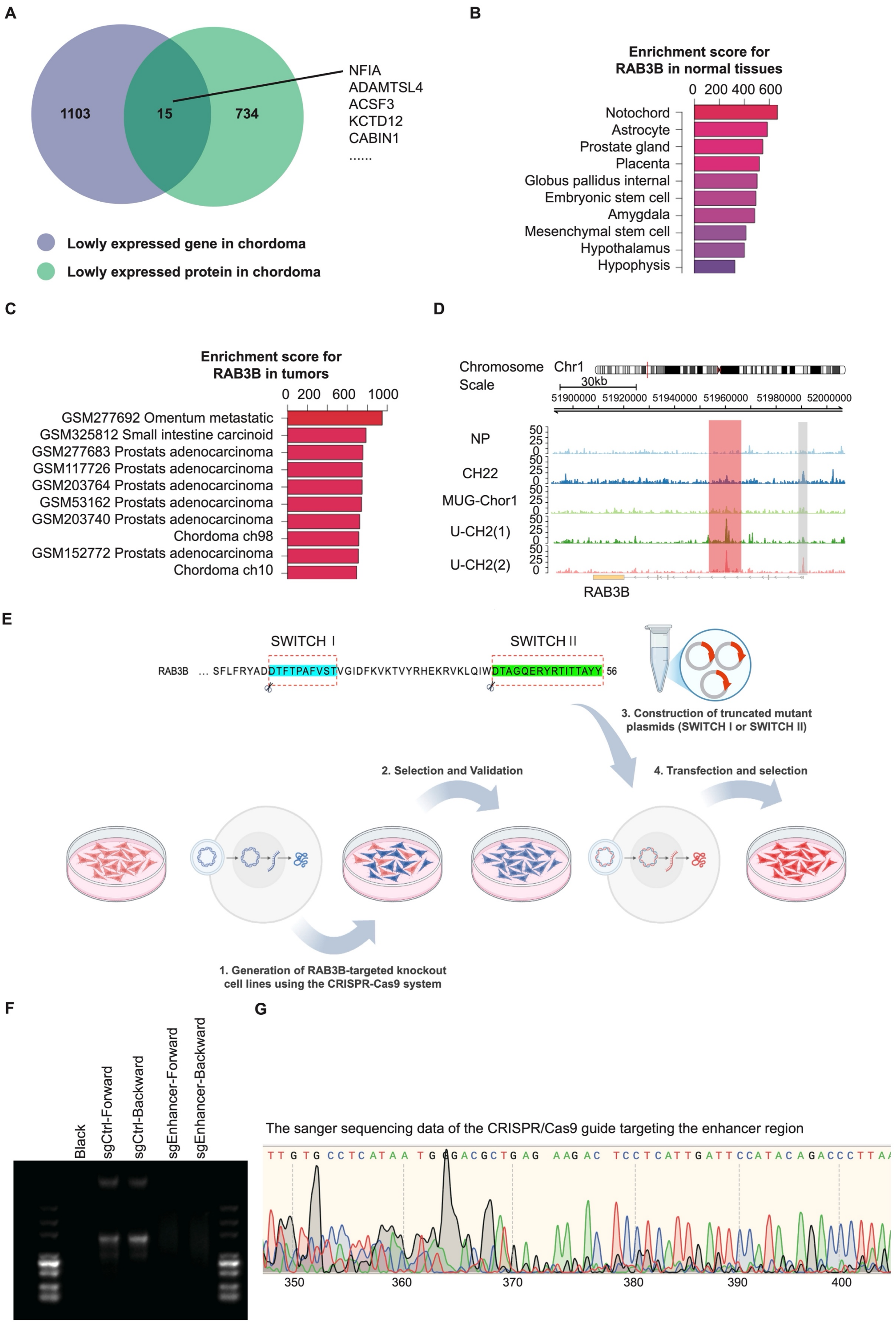

Figure S3. Enhancer-associated high transcriptional activity of RAB3B in chordoma

(A) The overlapped markers lowly expressed in chordoma. Enrichment score for RAB3B in normal tissues (B) and tumors (C) in the Chordoma Project Supplementary Material; (D) ATAC-seq data of

chordoma cell lines (CH22, MUG-Chor1 and U-CH2) and NP cells revealed the peaks in the region of RAB3B; (E) The schematic diagram of methodology for the deletion of the Switch I and Switch II domains in the RAB3B protein; (F) DNA gel electrophoresis of CH22 cells infected with or without sgCtrl or sgRAB3B-Enhancer; (G) The sanger sequencing data of the CRISPR/Cas9 guide targeting the enhancer region.

Abbreviations: ATAC-seq, Assay for transposable accessible chromatin by high-throughput sequencing; Ctrl, Control.

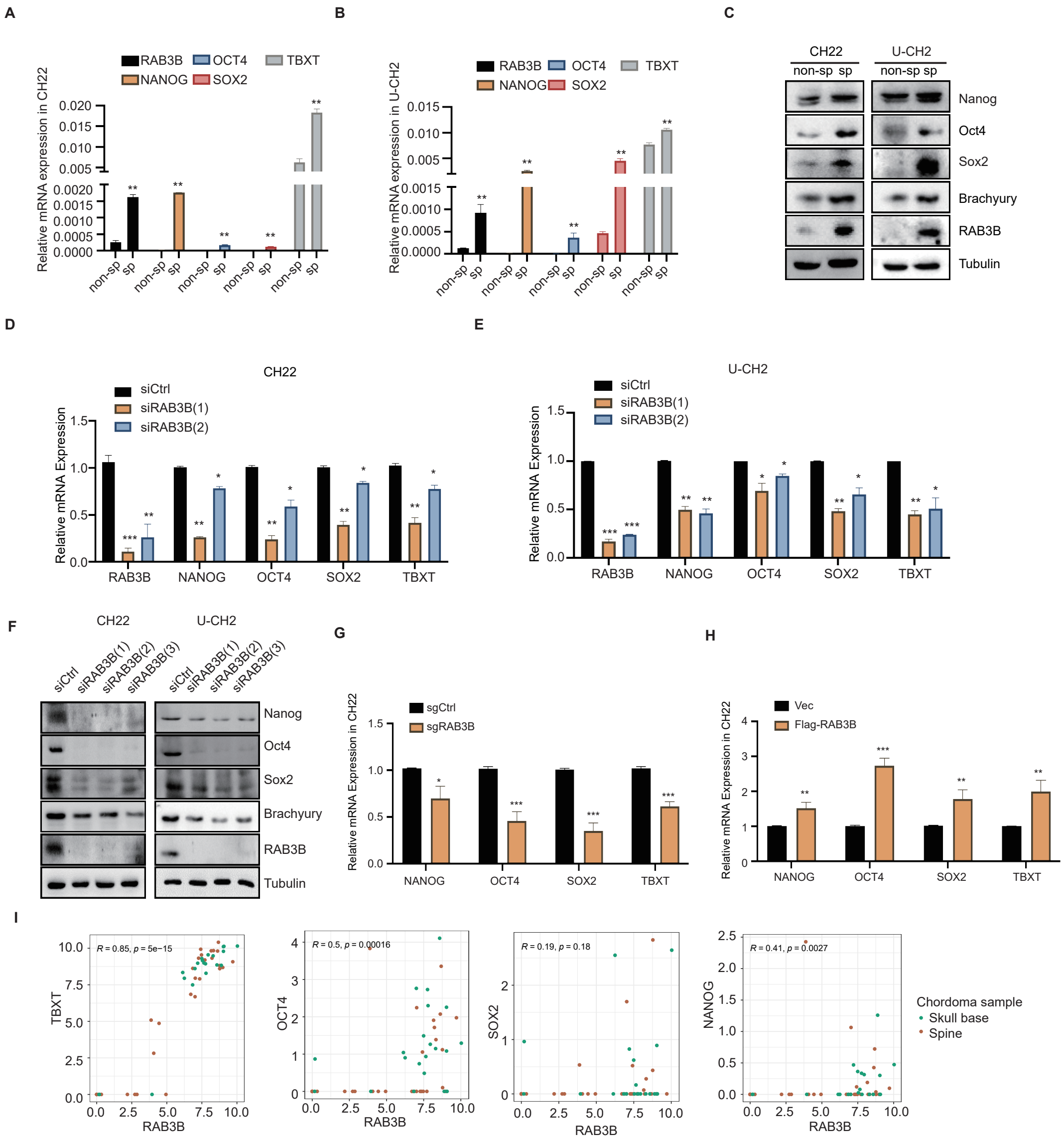

Figure S4. RAB3B enhances chordoma stemness and tumorigenic capacity

mRNA levels of stemness markers NANOG, OCT4, SOX2 and TBXT, along with RAB3B in CH22 (A) and U-CH2 (B) spheres and nonsphere cells; (C) Protein levels of stemness markers NANOG, OCT4, SOX2 and TBXT (brachyury), along with RAB3B in CH22 and U-CH2 spheres and nonsphere cells; mRNA levels of stemness markers in the RAB3B siRNA-treated CH22 (D) and U-CH2 (E) cells; (F) Protein levels of stemness markers in the RAB3B siRNA-treated chordoma cells; (G) mRNA levels of stemness markers in the CH22^{sgCtrl} and CH22^{sgRAB3B} cells; (H) mRNA levels of stemness markers in the CH22^{Vec} and CH22$^{Flag\text{-}RAB3B}$ cells; (I) The correlation between RAB3B and stemness marker TBXT, OCT4, SOX2 and NANOG based on the transcriptome data of chordoma. *P < .05, **P < .01, ***P < .001.

Abbreviations: siRNA: small-interfering RNA; Ctrl, Control.

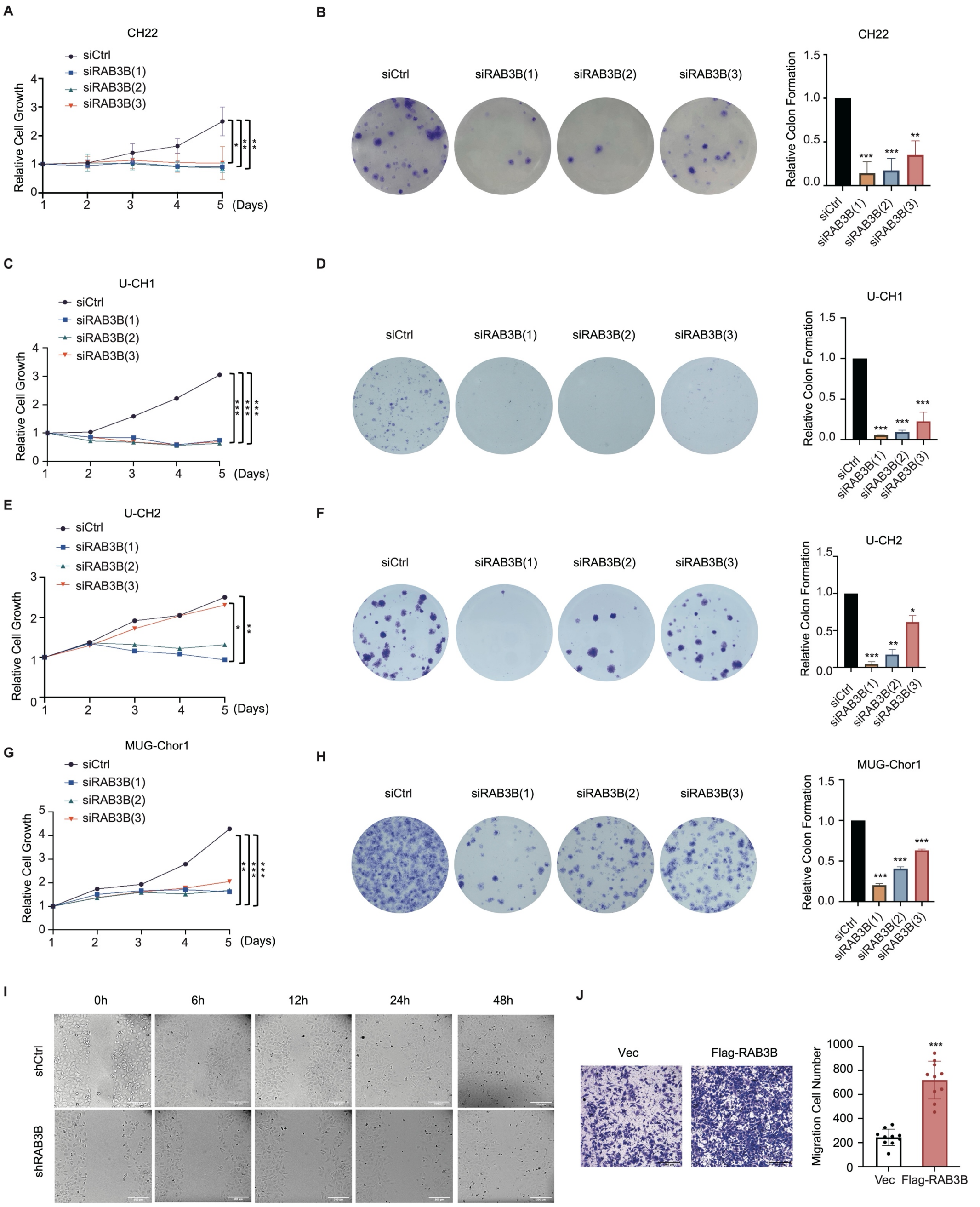

Figure S5. RAB3B mediates tumorigenic nature of chordoma

(A) MTT assay showed a significant time-dependent inhibition of cell proliferation after RAB3B siRNA treatment in CH22 cells. (B) Representative images of chordoma cell colony formation after treatment with RAB3B siRNA (left); Number of cell colony formation after the treatment of RAB3B siRNA in CH22 cells (right); (C) MTT assay showed a significant time-dependent inhibition of cell proliferation after RAB3B siRNA treatment in U-CH1 cells. (D) Representative images of chordoma cell colony formation after treatment with RAB3B siRNA (left); Number of cell colony formation after the treatment of RAB3B siRNA in U-CH1 cells (right); (E) MTT assay showed a significant time-dependent inhibition

of cell proliferation after RAB3B siRNA treatment in U-CH2 cells. (F) Representative images of chordoma cell colony formation after treatment with RAB3B siRNA (left); Number of cell colony formation after the treatment of RAB3B siRNA in U-CH2 cells (right); (G) MTT assay showed a significant time-dependent inhibition of cell proliferation after RAB3B siRNA treatment in MUG-Chor1 cells. (H) Representative images of chordoma cell colony formation after treatment with RAB3B siRNA (left); Number of cell colony formation after the treatment of RAB3B siRNA in MUG-Chor1 cells (right); (I) Representative images of chordoma cell migration in $CH22^{shCtrl}$ and $CH22^{shRAB3B}$ cells in wound healing assay; (J) Representative images of chordoma cell migration (left) and migration cell number (right) after overexpression of RAB3B in CH22 cells in transwell assay. *$P < .05$, **$P < .01$, ***$P < .001$. Abbreviations: siRNA: small-interfering RNA; Ctrl, Control.

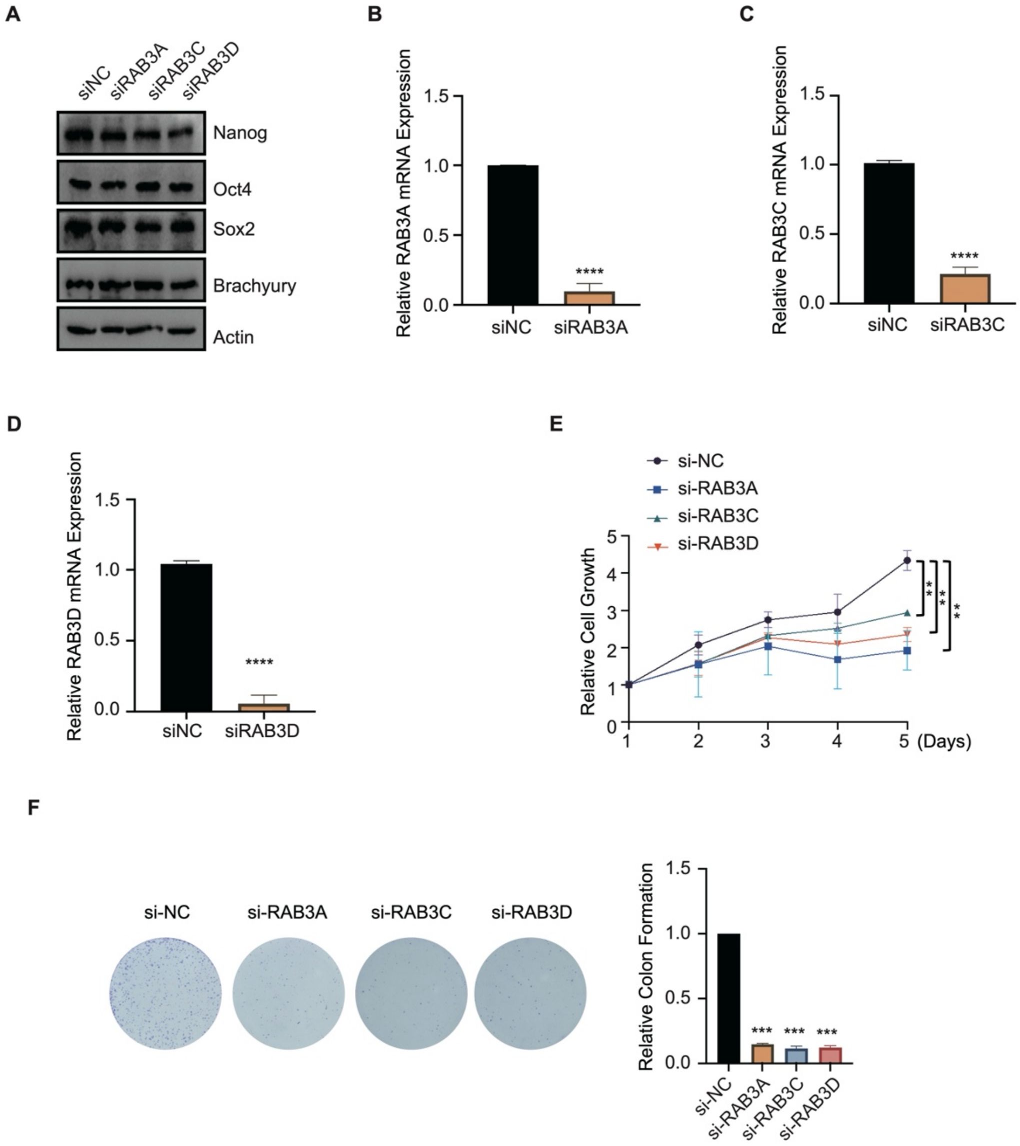

Figure S6. Roles of RAB3 members in the chordoma stemness and tumorigenesis

(A) Protein levels of stemness markers in the RAB3A/C/D siRNA-treated chordoma cells; mRNA levels of stemness markers in the RAB3A (B), RAB3C (C) and RAB3D (D) siRNA-treated CH22 cells; (E) MTT assay showed a significant time-dependent inhibition of cell proliferation after RAB3A/C/D siRNA treatment in CH22 cells. (F) Representative images of chordoma cell colony formation after treatment with RAB3A/C/D siRNA (left); Number of cell colony formation after the treatment of RAB3A/C/D siRNA in CH22 cells (right). $^{**}P < .01$, $^{***}P < .001$, $^{****}P < .0001$.

Abbreviations: siRNA: small-interfering RNA.

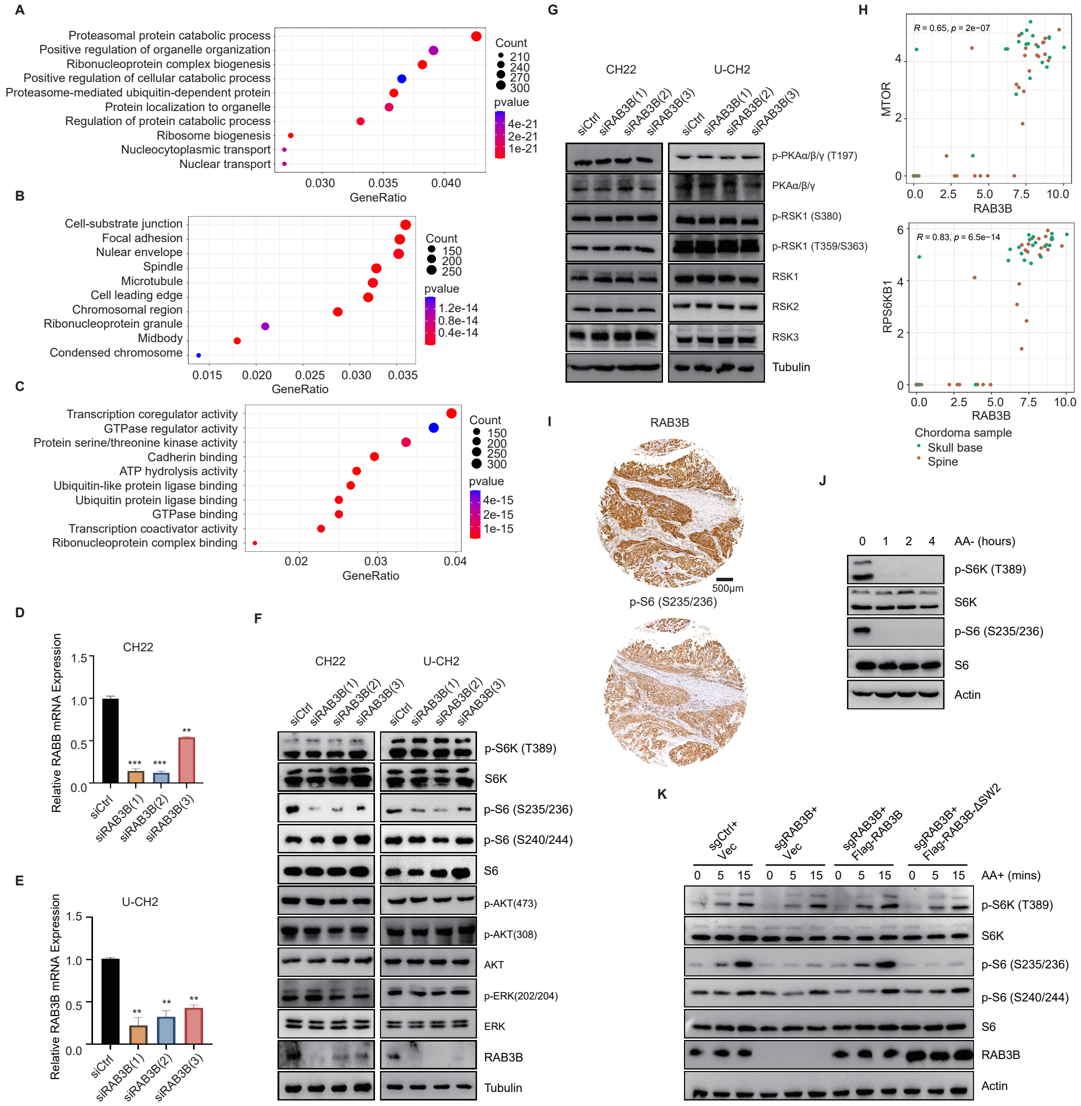

Figure S7. RAB3B regulates mTORC1 signaling via controlling site-specific S6 phosphorylation (S235/236)

GO analysis for BP (A), CC (B) and MF (C) of DEGs between RAB3B wildtype and knockdown CH22 cells; qRT-PCR analysis to assess the mRNA levels of RAB3B after RAB3B siRNA treatment in CH22 (D) and U-CH2 (E) cells. WB analysis to assess the protein levels of PI3K-AKT-mTOR signaling (F) and phosphorylase kinases of S6 (G) after RAB3B siRNA treatment in chordoma cell lines; (H) The correlation between RAB3B and MTOR/RPS6K1 based on the transcriptome data of chordoma; (I) Representative images of RAB3B and p-S6 (S235/236) based on the TMA of chordoma. Scale bar, 500 μm; (J) WB analysis to assess the protein levels of mTORC1 signaling under AA starvation; (K) WB analysis to assess the protein levels of mTORC1 signaling in RAB3B knockout, rescue and GTPase (ΔSW2) deletion CH22 cells under AA stimulation. **P < .01, ***P < .001.

Abbreviations: GO, Gene oncology; BP, biological process; CC, cellular component; MF, molecular function; DEGs, differentially expressed genes; qRT-PCR, Quantitative reverse transcription polymerase chain reaction; siRNA: small-interfering RNA; TMA, Tissue microarray.

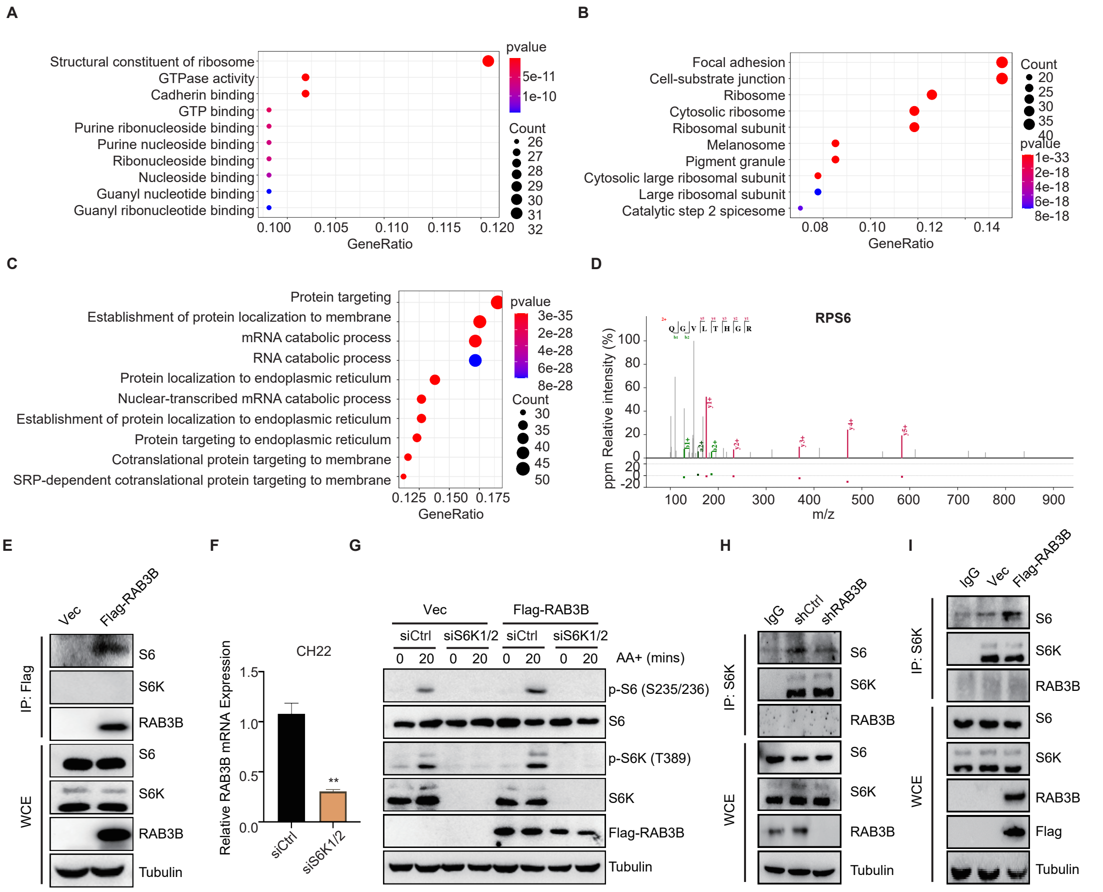

Figure S8 RAB3B directly works with S6 and potentiates S6K-mediated phosphorylation of S6

GO analysis of potential RAB3B-regulated proteins identified by MS regarding BP (A), CC (B) and MF (C); (D) Relative intensity (%) of RPS6 in MS identified by pFind protein identification system; (E) Co-IP assay to test the interaction between RAB3B and S6; (F) The relative mRNA expression of RAB3B in S6K1/2 siRNA-treated CH22 cells; (G) WB analysis to assess the protein levels of mTORC1 signaling in CH22^{Vec} and CH22$^{Flag\text{-}RAB3B}$ cells with or without the treatment of siS6K1/2 under AA stimulation; (H) Co-IP assay to test the interaction between S6 and S6K in CH22^{shCtrl} and CH22^{shRAB3B} cells; (I) Co-IP assay to test the interaction between S6 and S6K in CH22^{Vec} and CH22$^{Flag\text{-}RAB3B}$ cells. **P < .01.

Abbreviations: GO, Gene oncology; MS, mass spectrometry; BP, biological process; CC, cellular component; MF, molecular function; siRNA: small-interfering RNA; WB, Western blot; Ctrl, Control; Vec, Vector; AA, amino acid; Co-IP, Co-Immunoprecipitation;

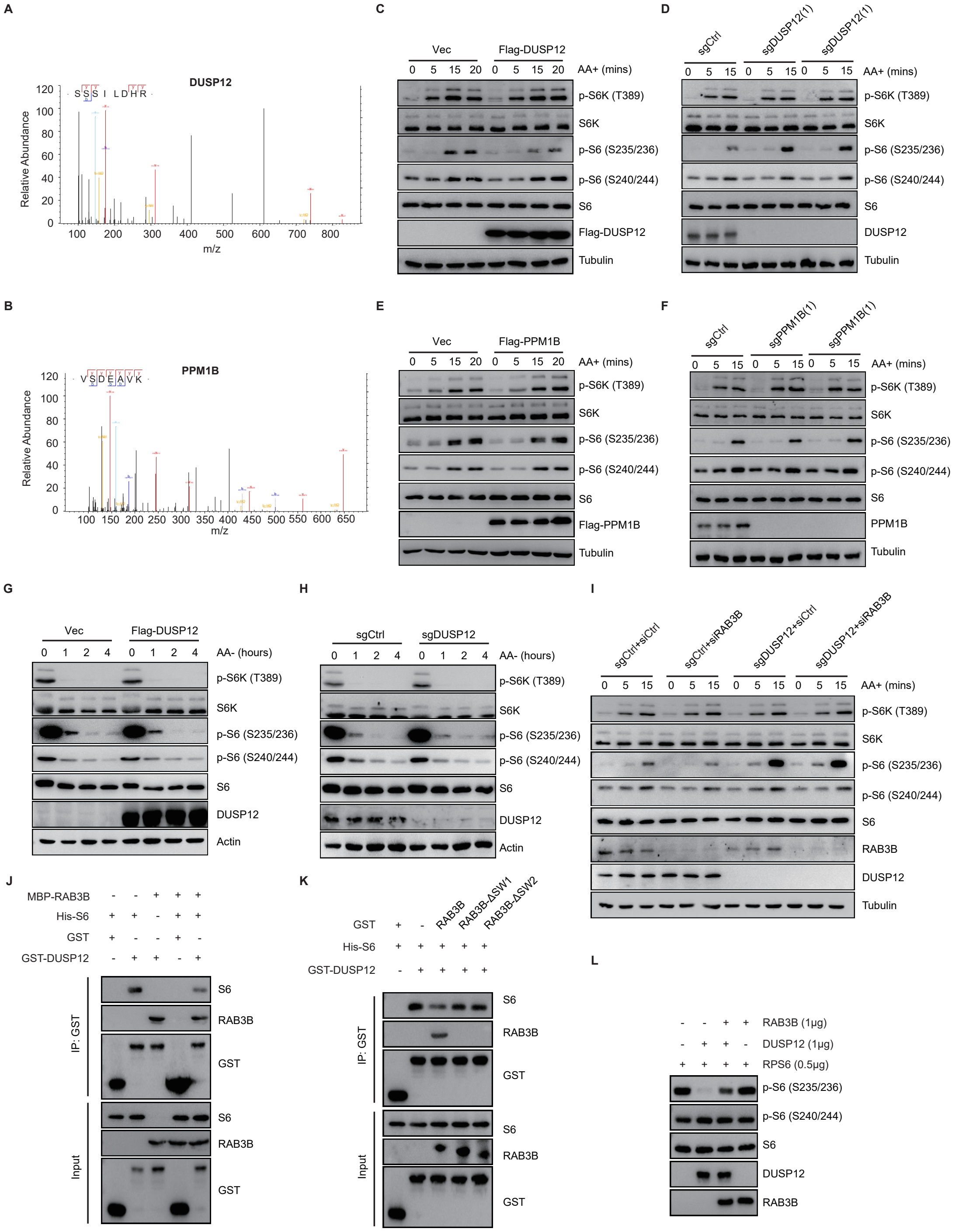

Figure S9 DUSP12 but not PPM1B regulates the dephosphorylation of p-S6

Relative abundance of DUSP12 (A) and PPM1B (B) in MS; (C) WB analysis to assess the protein levels of mTORC1 signaling in CH22^{Vec} and CH22$^{Flag\text{-}DUSP12}$ cells under AA stimulation; (D) WB analysis to assess the protein levels of mTORC1 signaling in CH22^{sgCtrl} and CH22^{sgDUSP12} cells under AA stimulation; (E) WB analysis to assess the protein levels of mTORC1 signaling in CH22^{Vec} and CH22$^{Flag\text{-}PPM1B}$ cells under AA stimulation; (F) WB analysis to assess the protein levels of mTORC1 signaling in CH22^{sgCtrl}

and CH22^{sgPPM1B} cells under AA stimulation. (G) WB analysis to assess the protein levels of mTORC1 signaling in CH22^{Vec} and CH22$^{Flag\text{-}DUSP12}$ cells under AA starvation; (H) WB analysis to assess the protein levels of mTORC1 signaling in CH22^{sgCtrl} and CH22^{sgDUSP12} cells under AA starvation; (I) WB analysis to assess the protein levels of mTORC1 signaling in CH22^{sgCtrl} and CH22^{sgDUSP12} cells with or without the treatment of siRAB3B under AA stimulation; (J) GST pull-down assay to show interaction between DUSP12 and S6 with or without RAB3B involvement; (K) GST pull-down assay to show the interaction between DUSP12 and RPS6 in RAB3B protein of GTPase (ΔSW1 or ΔSW2) deletion (Eukaryotic protein expression and purification); (L) An *in vitro* dephosphorylation assay to show the phosphorylated effects of RAB3B and DUSP12 in pS6.

Abbreviations: MS, mass spectrometry; WB, Western blot; Ctrl, Control; Vec, Vector; AA, amino acid.

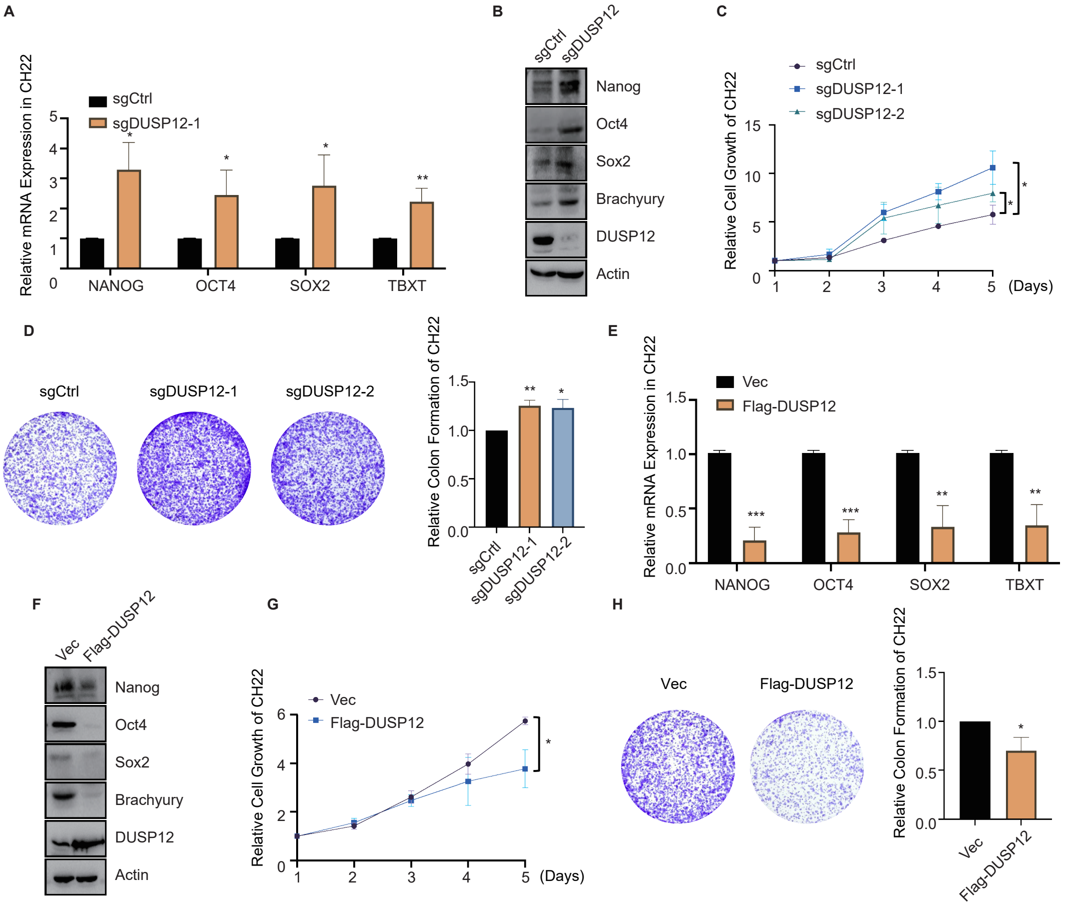

Figure S10 DUSP12 regulates the chordoma stemness and tumorigenesis

mRNA (A) and protein (B) levels of stemness markers in the CH22^{sgCtrl} and CH22^{sgDUSP12} cells; (C) MTT assay to evaluate the cell proliferation in the CH22^{sgCtrl} and CH22^{sgDUSP12} cells; (D) Representative images (left) and number (right) of chordoma cell colony formation in the CH22^{sgCtrl} and CH22^{sgDUSP12} cells; mRNA (E) and protein (F) levels of stemness markers in the CH22^{Vec} and CH22$^{Flag\text{-}DUSP12}$ cells; (G) MTT assay to evaluate the cell proliferation in the CH22^{Vec} and CH22$^{Flag\text{-}DUSP12}$ cells; (H) Representative images (left) and number (right) of chordoma cell colony formation in the CH22^{Vec} and CH22$^{Flag\text{-}DUSP12}$ cells. *P < .05, **P < .01, ***P < .001.

Abbreviations: Ctrl, Control; Vec, Vector.

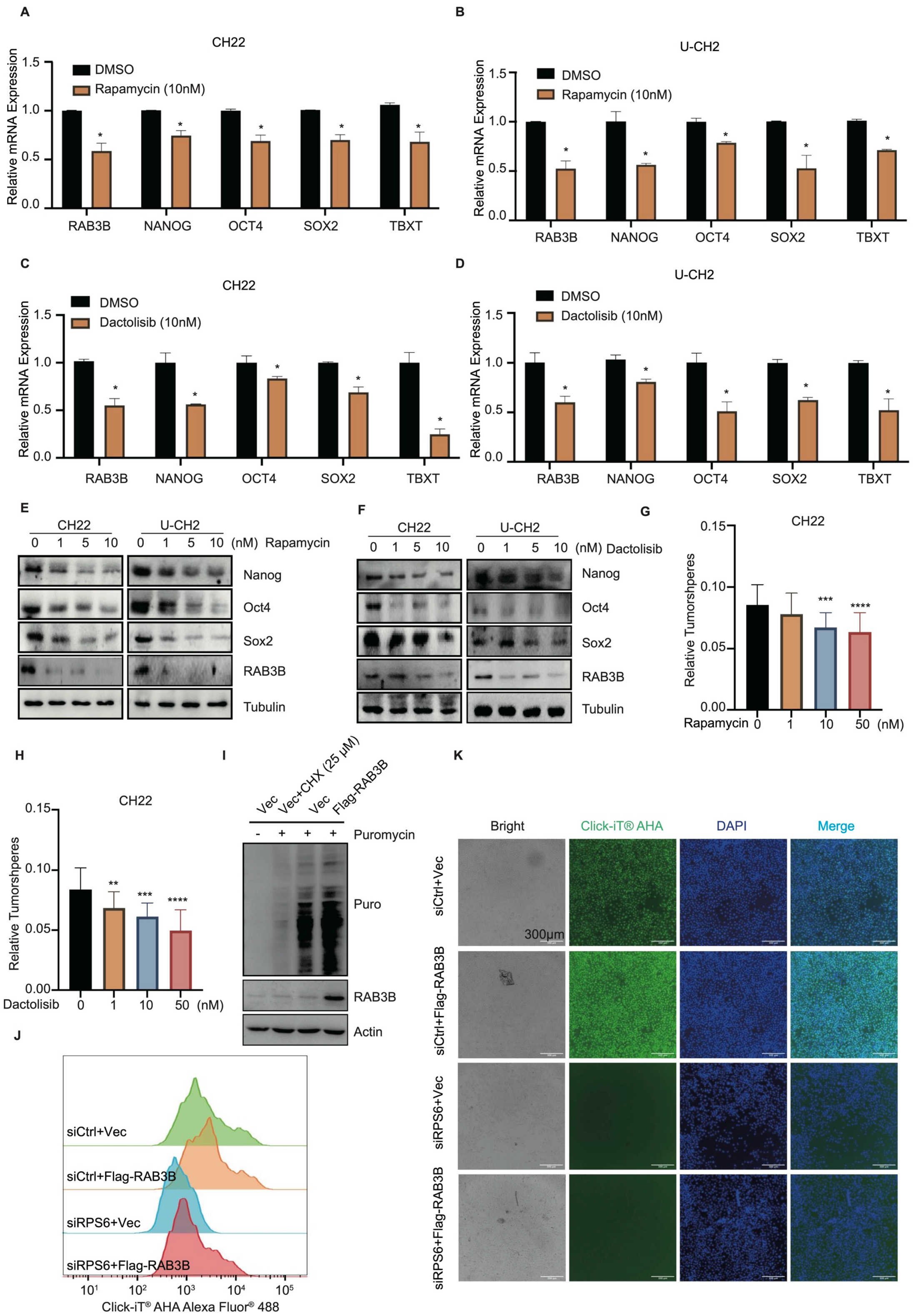

Figure S11 RAB3B regulates the cancer stemness and protein translation through S6

mRNA levels of stemness markers NANOG, OCT4, SOX2 and TBXT, along with RAB3B in CH22 (A) and U-CH2 (B) cells after rapamycin treatment; mRNA levels of stemness markers NANOG, OCT4, SOX2 and TBXT, along with RAB3B in CH22 (C) and U-CH2 (D) cells after dactolisib treatment;

Protein levels of stemness markers (Nanog, Oct4 and Sox2) in CH22 and U-CH2 cells with the treatment of rapamycin (E) and dactolisib (F); Numbers of CH22 tumorspheres after the treatment of rapamycin (G) and dactolisib (H); (I) The protein synthesis in $CH22^{Vec}$ and $CH22^{Flag\text{-}RAB3B}$ cells with or without CHX treatment in SUnSET assay; The protein synthesis in $CH22^{Vec}$ and $CH22^{Flag\text{-}RAB3B}$ cells with or without siRPS6 treatment in the Click-iT® AHA Alexa Fluor® 488 Protein Synthesis HCS Assay revealed by the flow cytometry (J) and fluorescence microscopy (K). *P < .05, **P < .01, ***P < .001, ****P < .0001.

Abbreviations: Ctrl, Control; Vec, Vector; AA, amino acid.

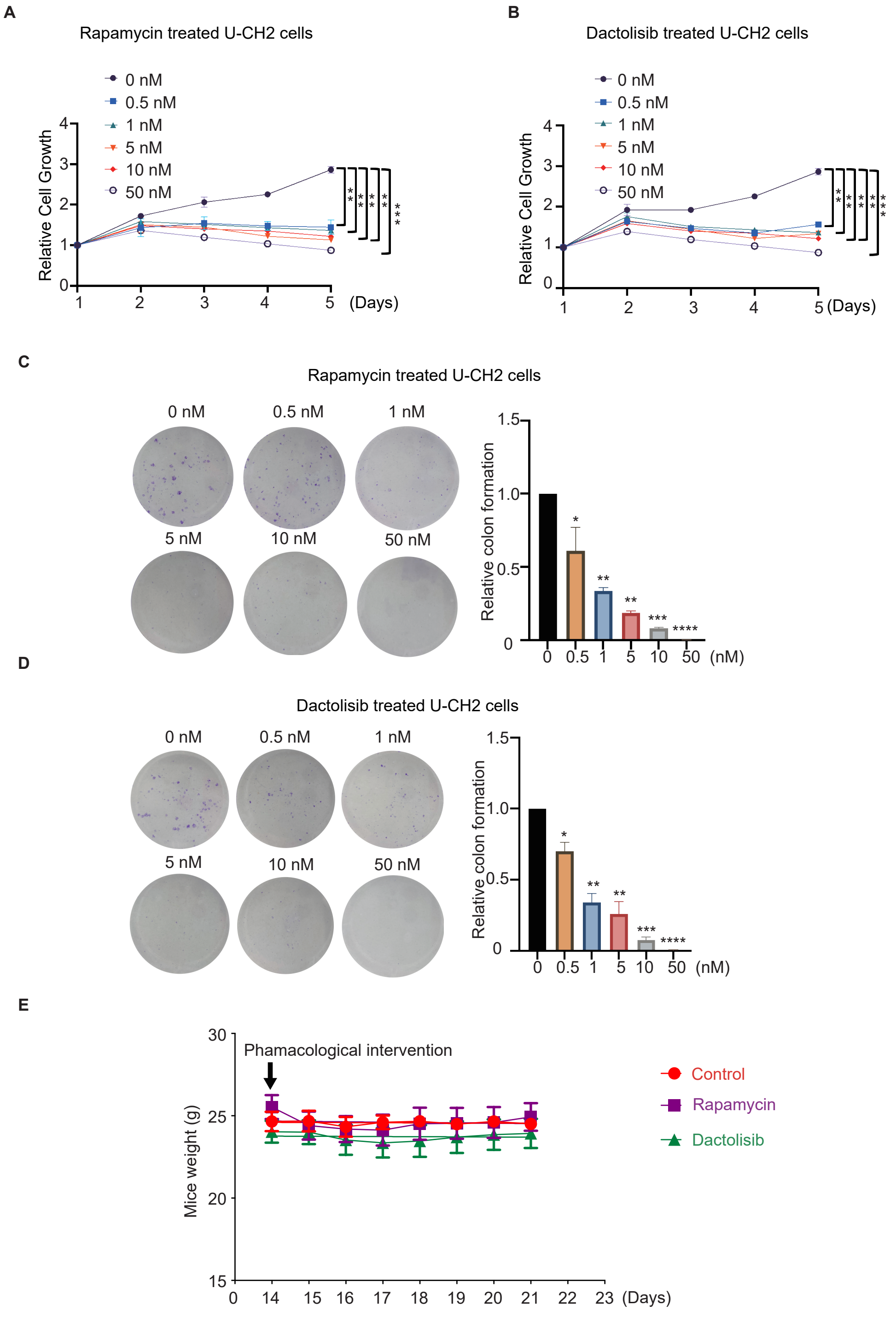

Figure S12 Targeting mTORC1 signaling is a potential therapeutic option for chordoma

MTT assay showed a significant inhibition of cell proliferation after rapamycin (A) and dactolisib (B) treatment in U-CH2 cells in a time- and dose-dependent manner; Representative images (left) and relative colon formation (right) of U-CH2 colony formation after rapamycin (C) and dactolisib (D) treatment; (E) Mice weights of PBS-, rapamycin- and dactolisib-treated CH22-bearing nude mice. *P < .05, **P < .01, ***P < .001, ****P < .0001.

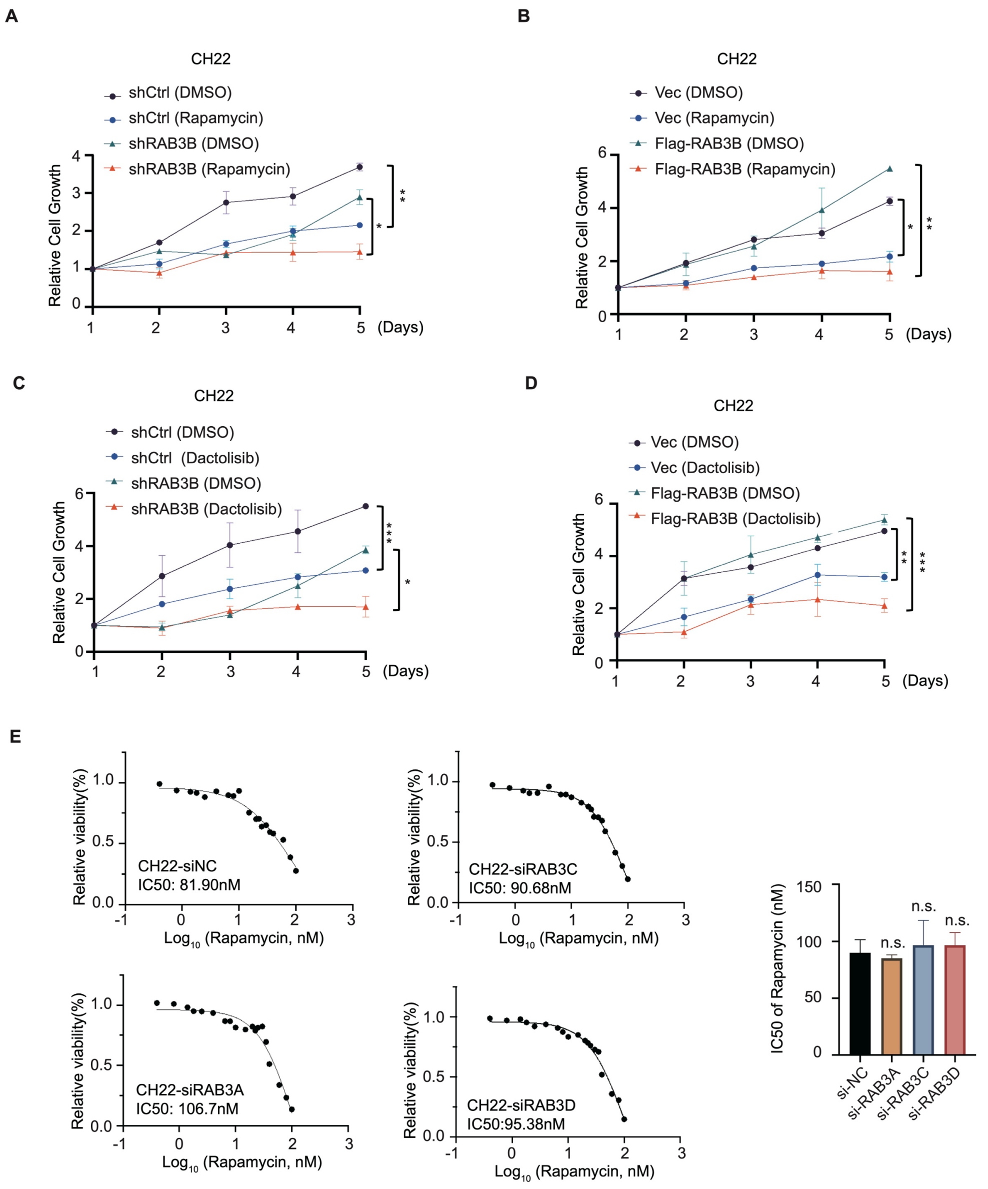

Figure S13 RAB3B/S6 axis indicates the drug susceptibility of mTORC1 inhibitors for chordoma

MTT assay of rapamycin for RAB3B wildtype and knockdown (A)/ overexpression (B) CH22 cells; MTT assay of dactolisib for RAB3B wildtype and knockdown (C)/ overexpression (D) CH22 cells; (E) IC50 of rapamycin in CH22 cells treated with RAB3A, RAB3C and RAB3D. *P < .05, **P < .01, ***P < .001.

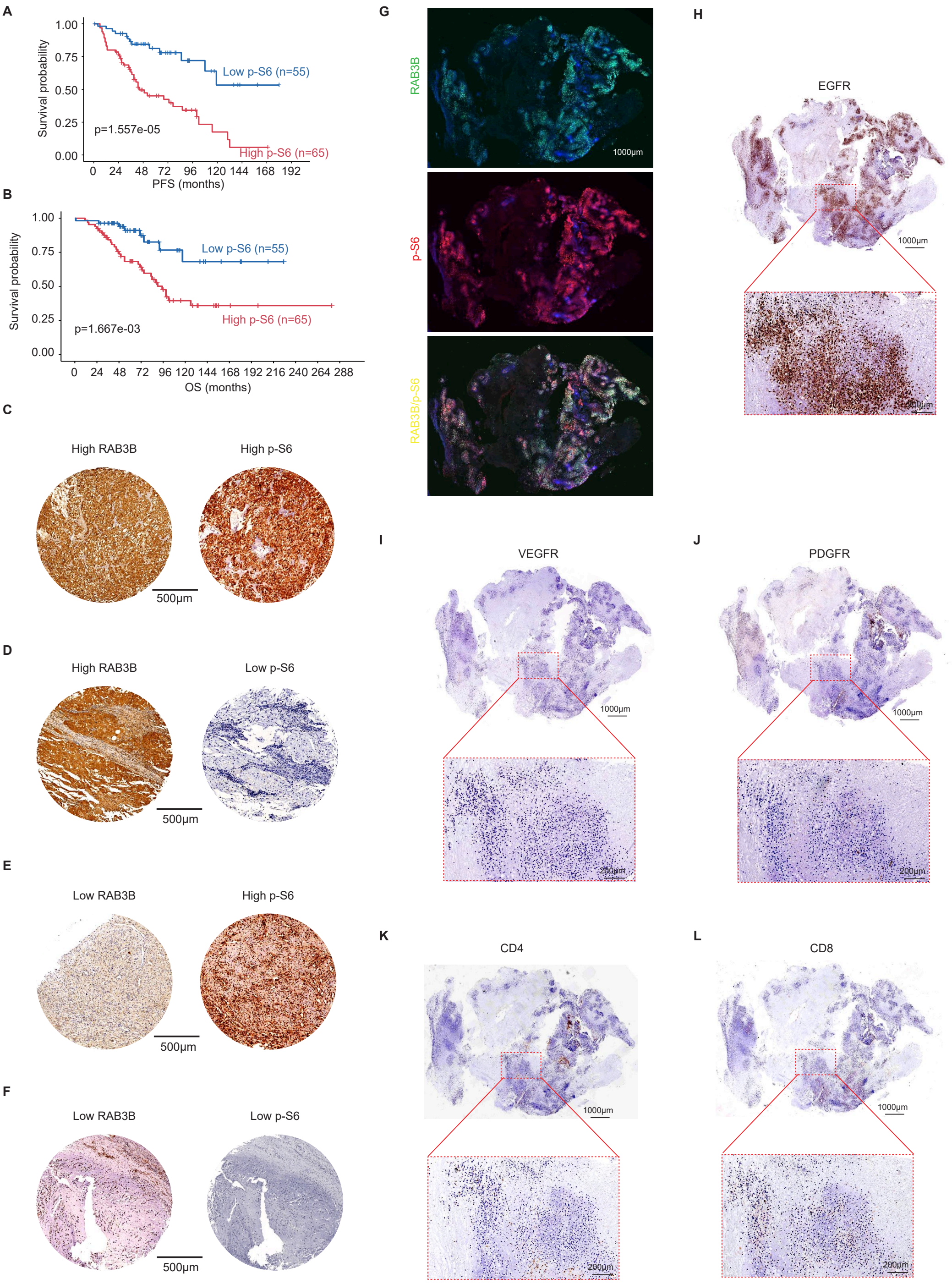

Figure S14. The clinical application of RAB3B/S6 axis in prognostic prediction and response to mTORC1-targeted therapy in chordoma patients

Kaplan-Meier analysis of PFS (A) and OS (B) of chordoma patients in low and high p-S6 (S235/236) group; The representative images of IHC in high RAB3B high p-S6 (S235/236) group (C), high RAB3B low p-S6 (S235/236) group (D), low RAB3B high p-S6 (S235/236) group (E) and low RAB3B low p-S6 (S235/236) group (F); (G) Immunofluorescence co-localization analysis of RAB3B and p-S6

(S235/236) in this chordoma case; The IHC of EGFR (H), VEGFR (I), PDGFR (J), CD4 (K) and CD8 (L) in this advanced chordoma case. Scale bar, 200 and 1000 μm.

Abbreviations: IHC, Immunohistochemistry; PFS, progression-free survival; OS, Overall survival.
